# Nutrition and Physical Activity Education in Medical School: A Narrative Review

**DOI:** 10.3390/nu16162809

**Published:** 2024-08-22

**Authors:** Joana Rodrigues Sousa, Vera Afreixo, Joana Carvalho, Paula Silva

**Affiliations:** 1 Laboratory of Histology and Embryology, Institute of Biomedical Sciences Abel Salazar (ICBAS), Rua de Jorge Viterbo Ferreira n.° 228, 4050-313 Porto, Portugal; joanaaguiarsousa@gmail.com; 2 Center for Research & Development in Mathematics and Applications (CIDMA), Department of Mathematics, University of Aveiro, 3810-193 Aveiro, Portugal; vera@ua.pt; 3 Research Centre in Physical Activity, Health and Leisure of University of Porto, 4200-450 Porto, Portugal; mjoanacarvalho@reit.up.pt; 4 Laboratory for Integrative and Translational Research in Population Health (ITR), 4050-600 Porto, Portugal; 5 iNOVA Media Lab, ICNOVA-NOVA Institute of Communication, NOVA School of Social Sciences and Humanities, Universidade NOVA de Lisboa, 1069-061 Lisbon, Portugal

**Keywords:** exercise promotion, medical student perceptions, active lifestyle, healthcare promotion, curriculum design, health diet, nutrition

## Abstract

This review explores the diverse landscape of integrating nutrition and physical activity education into medical school curricula, focusing on the imperative role of physicians in promoting health through lifestyle changes. By examining global medical education structures, we uncovered disparities in nutrition and physical activity training, and highlighted the need for a shared framework to address international and regional challenges. Despite acknowledging the importance of both nutrition and physical activity, studies have consistently uncovered deficiencies in medical school curricula, especially in skills related to providing lifestyle advice and behavioral counseling. Survey studies among medical students have illuminated various perceptions and knowledge gaps, emphasizing the need for more comprehensive and mandatory nutrition and physical activity training. While acknowledging progress, challenges, such as time constraints, resource availability, and faculty expertise, persist. Integrating lifestyle education results in resistance, a demand for strategic communication, and faculty buy-ins. These findings underscore the importance of a holistic approach that balances theoretical knowledge, practical skills, and confidence that medical students need to promote effective nutrition and physical activity in healthcare.

## 1. Introduction

### 1.1. Promoting Health: The Impact of Diet and Physical Activity Adherence

The significance of a healthy lifestyle, including adequate diet and physical activity (PA), has been recognized worldwide. A healthy lifestyle is characterized by a balanced diet, regular PA, and overall well-being, which are essential for maintaining health, enhancing physical fitness, and reducing the risk of chronic diseases [1]. Adequate nutrition supports physical fitness and recovery processes, whereas PA is crucial for preventing disease and managing stress [2]. Diet and PA must be considered as interrelated components of a holistic approach to preventive medicine and health promotion. In the past decade, the World Health Organization (WHO) has emphasized the crucial role of health systems in promoting diet and PA to combat noncommunicable diseases (NCDs) [3,4]. The WHO’s objectives to increase adherence to a healthy diet are multifaceted and include the promotion of guidelines to prevent chronic diseases, development of responsive health systems, and integration of health considerations into broader policy areas. These objectives are pursued with an understanding of the varying levels of adherence across different regions and socioeconomic contexts and through targeted efforts to address specific nutritional challenges such as obesity, NCDs, and maternal and child health [5]. In 2018, the World Health Assembly (WHA) approved a new Global Action Plan on Physical Activity (GAPPA) aimed at reducing the prevalence of physical inactivity by 15% by 2030. The WHO recommends that all countries establish national guidelines and set PA targets to help support populations achieve these targets and maintain healthy levels of PA [6].

Interestingly, while the importance of these lifestyle factors is widely recognized, adherence to the recommended dietary and exercise guidelines is not universal. This highlights the gap between knowledge and practice in the adoption of healthy lifestyle behaviors. Recent global statistics have highlighted a significant discrepancy between dietary and PA recommendations, and actual habits among adults. Studies have shown that a substantial portion of the adult population does not adhere to the dietary guidelines that are essential for health and well-being. For instance, WHO reports that many adults fail to consume the recommended daily intake of 400 g of fruits and vegetables, limit the total fat intake to less than 30% of the total energy, and maintain salt consumption below 5 g per day [4]. In the United States, the Healthy Eating Index scores reveal that the average diet quality score for adults is 58 out of 100, indicating a significant gap between the actual diet and recommended nutritional guidelines. These scores are derived from data collected through the National Health and Nutrition Examination Survey (NHANES) and reflect the widespread challenges in meeting dietary recommendations [7]. Recent studies have shown that adherence to dietary recommendations among European adults varies significantly across regions and demographics. A study utilizing data from the United Kingdom (UK) Biobank found that nearly one-third of participants did not meet any dietary recommendations, whereas only 9.5% adhered to three or four dietary guidelines. This indicates a substantial gap in dietary guidelines aimed at reducing the risk of chronic diseases and improving overall health outcomes. The study highlighted that better adherence was associated with lower risks of all-cause mortality and cardiovascular disease [8]. Regarding PA, in the WHO revised recommendations published in 2020, adults are advised to engage in a minimum of 150–300 min of moderate-intensity PA or 75–150 min of vigorous-intensity PA per week [3]. Alternatively, adults can choose an equivalent combination of aerobic PA each week [3]. However, recent global statistics show that 1.4 billion adults, constituting 27.5% of the worldwide adult population, fail to meet the recommended PA level essential for improving and protecting their health and well-being [9,10].

The challenges of maintaining a healthy lifestyle are also evident among university students, as demonstrated in a study by Moscatelli et al. (2023). This study assessed the lifestyle, eating habits, and effects of nutritional education among undergraduate students in Southern Italy. The findings revealed significant improvements in blood pressure, PA levels, and dietary habits following a six-month period of training seminars focused on nutrition and lifestyle education. These results underscore the importance of targeted educational interventions to promote healthier behaviors, particularly among young adults transitioning to independent living [11]. Recent studies have highlighted the positive impact of comprehensive lifestyle education on medical students. For instance, Jones et al. (2023) discussed the integration of nutrition education into the medical curriculum, emphasizing the need for such education to be interwoven across various medical disciplines to improve overall healthcare outcomes [12]. Blythe et al. (2022) review innovations in nutrition education within UK medical schools, illustrating the benefits of creating dedicated roles for nutrition education to enhance the visibility and effectiveness of such programs [13]. Rocliffe et al. (2024) examined the impact of physical education and sports on adolescent mental health and well-being, underscoring the necessity of incorporating PA into educational curricula to address broader health challenges [14]. It is highly recognized that the widespread absence of formal nutrition and PA education in medical schools has persisted over many years. A comparative analysis of the specific credits and skills in nutrition and PA offered by non-European (EU) and EU universities for the formation of medical doctors revealed significant differences influenced by educational policies and healthcare priorities. This comparison is grounded in the evaluation of curriculum structures, credit allocations, and emphasis on interdisciplinary training. In the EU, medical education is structured under frameworks such as the Bologna Process, which standardizes higher education across member states. Generally, EU universities integrate nutrition and PA into their medical curricula through both mandatory and elective courses. Studies indicate that EU universities typically allocate around 20 to 30 credits to nutrition education, extensively covering clinical and public health nutrition. Additionally, PA education is significantly included within public health curricula, emphasizing exercise physiology and lifestyle medicine [15]. In contrast, non-EU universities, especially in the United States (US), show greater variability and often less integration of these subjects. Research by Adams et al. (2015) notes that US medical schools average about 19.6 h of nutrition education throughout the medical curriculum, translating to fewer credits compared to EU counterparts. Similarly, PA education in non-EU universities is less integrated, with a primary focus on exercise prescription rather than a broader public health approach [16]. This disparity reflects differing educational philosophies and healthcare priorities. EU medical education tends to adopt a more holistic approach to patient care, acknowledging the significant role of lifestyle factors in disease prevention and management. In contrast, non-EU systems, particularly in the US, prioritize biomedical sciences, often at the expense of comprehensive nutrition and PA training. The structured and comprehensive approach in the EU ensures medical graduates are better equipped with essential skills in nutrition and PA, addressing the rising need for lifestyle medicine in managing NCDs. By contrast, the less consistent approach in non-EU universities underscores the need for global standardization to enhance the quality of medical education and patient care outcomes.

### 1.2. The Vital Role of Healthcare Settings in Promoting Diet and Physical Activity

The WHO recognizes the importance of promoting healthy diets within healthcare facilities as part of a broader strategy to tackle NCDs [17]. The WHO’s Health Promoting Hospitals network aims to reorient healthcare services toward a more health-oriented approach that includes the promotion of healthy diets among both patients and healthcare staff [18]. However, studies have shown that hospital management policies often fall short of supporting comprehensive health promotion strategies, including those aimed at improving dietary habits among patients [19]. These gaps in policy implementation can hinder efforts to promote healthy eating habits and reduce diet-related diseases. Additionally, environmental interventions in healthcare settings, such as changes in food supply and nutritional information at the point of choice, are part of the efforts to encourage positive nutritional behavior [20]. The Global Action Plan for Physical Activity Promotion (2018–2030), developed by the WHO, emphasizes the value of implementing PA promotion schemes within healthcare facilities, mainly oriented toward patients and conducted by healthcare professionals who have received suitable training [21]. A tailored toolkit has been created for primary care, which encompasses strategies to aid countries in implementing and supporting PA evaluation and counseling systems as part of universal healthcare [22]. In 2021, despite ongoing efforts, less than 50% of countries reported implementing a national protocol for the stated objective [10]. The 5A’s model, which comprises Ask, Advise, Assess, Assist, and Arrange, serves as a structured intervention approach designed to encourage increased PA levels among patients [22]. This model calls for clinicians to first inquire about patient adherence to recommended PA guidelines and evaluate any potential risks or restrictions. Subsequently, they should provide information on the health advantages of PA and collaborate with patients to establish targets for PA. Clinicians must then assist patients in recognizing obstacles that hinder their PA and developing strategies to tackle these obstacles [22]. Finally, they must arrange for follow-up support to ensure that patients receive continuous assistance in pursuit of increased PA levels. These models identify PA levels and sedentary behavior, provide verbal encouragement, evaluate physical fitness, prescribe personalized exercise programs, and refer patients to third-party services. These intervention models can be implemented individually or in combination to address patients’ PA needs in primary care settings [22].

The healthcare sector presents a unique opportunity to promote a healthy diet and PA across populations. First, it allows for direct contact with the large demographics of the population across their lifespan, as most individuals visit their healthcare providers at least once a year [23,24,25]. This enables the delivery of individualized care, including personalized risk assessments, tailored advice, and support for behavioral changes. Second, public trust in the healthcare sector and primary care physicians is considered to be the most adequate, reliable, and approachable source of nutritional information [26,27,28,29,30]. Despite the essential function of health care professionals in primary care and the documented success of interventions, the number of practitioners actively participating in healthy lifestyle promotion could potentially increase. Available data regarding the promotion of a healthy diet/PA in primary healthcare settings reveal disparities in assessment and counseling practices. The prevalence of dietary counseling in primary care varies, with studies indicating that a significant proportion of primary care physicians recognize the importance of diet and nutrition in health maintenance and disease prevention, yet often do not provide extensive dietary counseling [31]. Approximately 70% of primary care physicians in a German study reported routinely providing brief consultations on diet modifications to at least half of their patients [29]. Nearly all surveyed Croatian [32] and Lebanese [33] general practitioners claimed to offer nutritional support in everyday practice. However, only 20% of Croatian general practitioners provide counseling to all patients, with 80% targeting those with specific health risks [32]. A study in Saudi Arabia found that one-third of primary care doctors never or rarely provided nutritional counseling, while the other third did so only half of the time [34]. More than 90% of Australian general practitioners proactively discussed nutrition with patients, and nearly 80% stated that patients often initiated these conversations [35]. The prevalence of PA counseling in primary care is generally low (37.9%) [36]. The extent to which primary care practitioners screen for and provide advice on PA varies significantly (2.4–100% and 0.6–100%, respectively) [36,37].

Understanding the challenges and barriers faced by healthcare professionals in promoting healthy lifestyles is critical. Research has consistently shown that external factors, such as time constraints, patients’ lack of interest, and financial limitations, have a profound impact on diet and PA promotion [33,38,39,40,41]. These studies underscore the need to address these barriers to enhance the effectiveness of both nutrition and PA initiatives. Additionally, healthcare professionals often report significant gaps in their knowledge and skills related to promoting both diet [42,43,44] and PA [39,40,41,45,46], highlighting the urgent need for targeted educational programs to build their confidence and competence in this area. There is an urgent need for the integration of public health principles and consideration of behavioral and psychosocial factors within hospital-based healthcare systems. This study emphasizes the crucial role of integrating nutrition and PA counseling into medical school curricula to equip physicians with the skills necessary to effectively manage chronic diseases.

The primary objective of this review is to critically evaluate the current state of nutrition and PA education within the medical school curriculum. This encompasses examining the structural organization of PA education, identifying key components and content, and assessing the effectiveness and inclusivity of nutrition and PA education in medical school programs. Additionally, this review aims to provide insights from survey studies—quantitative or qualitative research that collects data through structured questionnaires or interviews conducted among medical students. This investigation included a comprehensive analysis of the research designs, methodologies, and sample characteristics of these surveys, as well as an evaluation of the perceptions, knowledge, attitudes, and practices of medical students concerning nutrition and PA education.

Our decision to write this review stems from the recognized need to address disparities in nutrition and PA education across medical schools globally. Despite the well-documented benefits of a healthy lifestyle in preventing chronic diseases and promoting overall well-being, a significant gap remains in how these subjects are integrated into medical curricula. By synthesizing existing research and providing a critical assessment, we aimed to highlight the areas requiring improvement and propose a framework for enhancing nutrition and PA education in medical training. This review is intended to inform educators, policymakers, and researchers about the current landscape and advocate for more comprehensive and standardized approaches to lifestyle education in medical schools.

## 2. Nutrition and Physical Activity Education in Medical School Curricula

### 2.1. Nutrition Education

The assessment of nutrition education within medical curricula reveals a shared understanding of its relevance but also underscores substantial deficiencies in its incorporation and efficacy. Medical students and educators acknowledge the vital function of nutrition in managing persistent illnesses and overall patient care; however, research has consistently unveiled dissatisfaction with the present level of nutrition education [47]. To rectify this issue, medical school curricula must be revised to prepare future general practitioners with indispensable knowledge and practical abilities. By incorporating nutritional education into undergraduate and graduate medical education, medical educators can prepare physicians to address the dietary requirements of their patients, ultimately enhancing the health outcomes of the population [48].

The inclusion of nutrition in medical curricula worldwide is not uniformly mandated, with significant variability in the presence and extent of nutrition education across different regions and schools. Lepre et al. (2021) [49] highlighted that only 44% of accreditation and curriculum guidance internationally includes nutrition, indicating an inadequate representation of nutrition in medical education at various levels. This lack of uniformity persists despite the recognized importance of diet in health outcomes and the role of physicians in providing dietary advice. Interestingly, while Chung et al. (2014) [15] reported that 68.8% of surveyed medical schools in Western European Union countries require some form of nutrition education, actual contact hours remain limited, averaging 23.68 h. This suggests that even among schools that include nutrition education, the depth and comprehensiveness of the training may be insufficient.

Over the past four decades, several initiatives have aimed to integrate nutrition into undergraduate medical education programs. For instance, the NIH-funded Nutrition Academic Award (1998–2005) helped develop nutrition education strategies and assessment tools, which improved nutritional knowledge and counseling confidence among medical students [50]. However, this program has not achieved widespread adoption or sustained integration into the medical curricula. Surveys of US medical schools have consistently shown a lack of adequate nutrition education, with many failing to provide the recommended minimum of 25 h of nutrition training [51]. A 2014 survey revealed that 71% of medical schools did not meet this minimum, and where nutrition curricula existed, they were mostly limited to preclinical courses, with little clinical application [16]. This challenge persists as medical advances continue to expand the required knowledge base, making it difficult to integrate essential content, such as nutrition. Unlike Ireland, the US, Canada, the UK, Australia, and New Zealand have visible curriculum guidelines for undergraduate nutritional education [35]. Guidelines in the US are detailed and prescription-oriented, whereas those in Australia and New Zealand emphasize the ability to identify nutritional risks. In contrast, the UK and US guidelines primarily focus on body weight assessment, potentially overlooking other important aspects of nutritional care [35]. Only the UK had mandatory nutritional guidelines for medical school curricula in 2015 [35]. Differences in curricular guidelines pose challenges to achieving a uniform curriculum and consistent nutritional counseling practices. The NNEdPro Global Institute for Food, Nutrition, and Health has demonstrated improvements in nutritional education in these countries. It emphasizes the importance of nutrition as part of a doctor’s responsibilities and promotes cooperation and consensus on best practices [52]. NNEdPro launched a nutrition education package as part of the Nutrition Education Policy in Healthcare Practice (NEPHELP)’ project. The aims of the NEPHELP were to (1) develop, evaluate, and implement nutrition education workshops and educational resources; (2) understand the feasibility and acceptability of a nutrition education model via participant and facilitator feedback; and (3) gain insights into where doctors and health professionals see the place for nutrition in their education. At Harvard Medical School, an innovative curriculum involving problem-based learning and simulated cases significantly improved students’ confidence in diet and exercise counseling [53]. This program highlighted the effectiveness of integrating interactive and practical learning methods into the curriculum. Similarly, the University of Nevada School of Medicine’s approach, which combined lectures with small-group discussions and CD-ROM programs, received high ratings from students and demonstrated above-average nutrition subscores [54].

In graduate medical education, nutrition education should build upon the foundation established during undergraduate medical education by tailoring the content to the specific needs of each medical specialty [55,56]. Although physicians do not require detailed nutrition training from a registered dietitian, they require a core foundation of evidence-based knowledge and practical skills to provide timely dietary advice and effectively collaborate with nutrition professionals. Studies have demonstrated that primary care [57,58,59], obstetrics [60,61], and surgery [62,63] residents frequently lack the preparation required to meet patients’ nutritional requirements. Enhancing nutrition education in graduate medical education can improve trainees’ confidence and capacity to execute nutritional counseling. Specialties such as cardiology and endocrinology naturally demand advanced nutrition knowledge owing to the dietary sensitivities of their patient populations. However, many cardiologists [55] and endocrinologists [64] have reported insufficient nutritional education during training. Similar gaps exist in gastroenterology fellowship programs, emphasizing the need for more standardized and comprehensive nutrition education across all specialties [65,66].

University of Arizona College of Medicine, with its extensive 75 h program, reported significant improvements in students’ objective structured clinical examination (OSCE) Nutrition Scores and received positive feedback regarding the integration of nutrition into the curriculum. This indicates a deeper and more comprehensive understanding of nutritional principles and better preparedness for applying this knowledge in clinical practice [67]. Determining the “best” method of instruction from the provided data can be challenging because each method has its own strengths and may work best in different contexts. However, some insights can be drawn from the reported outcomes of various programs. For instance, a combination of methods, particularly those that integrate nutrition education throughout the curriculum and include problem-based learning, workshops, and interactive components, tends to produce favorable outcomes [53,67,68]. The outcomes from these institutions demonstrate that students generally respond well to nutrition education, which is integrated, interactive, and comprehensive. Programs that effectively blend these elements tend to result in higher satisfaction, better preparedness, and greater ability to apply nutrition knowledge in clinical practice. Challenges across institutions include limited time within already packed medical curricula, varying levels of emphasis on nutrition, and insufficient faculty training in nutrition education. Furthermore, assessment methods vary, with some schools using OSCEs and others relying on self-reported confidence and knowledge surveys leading to inconsistencies in measuring outcomes. In the future, the integration of comprehensive and standardized nutrition education is essential. Enhanced training for faculty members, consistent assessment methods, and increased hours dedicated to nutrition within the curriculum are necessary. Developing a universally accepted framework for nutrition education in medical schools could help address these challenges, ensuring that future physicians are well prepared to effectively integrate nutrition into patient care (Table S1 in the Supplementary Material provides a detailed overview of the various nutrition education programs implemented in medical schools, with a focus on their methodologies, assessment techniques, and outcomes).

For practicing doctors, continuing medical education is crucial to address the existing gaps in knowledge and enable effective nutritional counseling. Health policy adjustments are required to motivate general practitioners to expand their knowledge in this area. Continued medical education programs in the US are leading nutrition training programs for practicing doctors [69]. Some examples are the Lifestyle Medicine course, Mayo Clinic Nutrition and Wellness in Health and Disease, and the Nutrition and Health Conference, which reviews the latest nutrition-related information [70]. Workshops have also improved the confidence of primary care professionals in nutritional counseling [71,72,73].

Overall, the findings of these studies advocate for a more structured, integrated, and experiential approach to nutrition education in medical training [74,75,76,77,78,79,80,81,82,83,84,85]. Addressing existing gaps and standardizing instructional methods and assessments are crucial steps toward better preparing future physicians to meet the nutritional needs of their patients, ultimately contributing to improved public health outcomes.

### 2.2. Physical Activity Education

Participation in regular PA is imperative to prevent NCDs and maintain overall well-being [23,86]. Physicians, as trusted sources of health information, play a pivotal role in encouraging PA among patients. This reality presents unprecedented challenges for the medical profession, imposing changes in public health strategies and fundamentally re-evaluating medical education. Studies conducted in different countries have highlighted the inadequacy of PA education in medical school curricula, highlighting a critical gap in physician training [87,88].

This issue persisted since the early recognition of the widespread absence of formal PA education in medical schools in 1975. A survey from that era indicated that only 16% of US medical schools included courses specifically addressing exercise as a component of preventive medicine [89]. These results were confirmed in a different study where it was found that only 13 out of 102 US allopathic medical schools provided instruction on the health benefits of PA and just six of these schools made it a mandatory part of the curriculum [90]. The topics covered during instruction were limited, with a focus on counseling patients to encourage PA [90]. Stoutenberg et al. (2015) [91] obtained different results from structured interviews with program directors of 74 of 171 accredited US medical education programs in 2013. The results showed that 78.4% of the programs included PA training in their curricula. However, a lower proportion of programs included essential tools for PA counseling, such as education on national recommendations for aerobic activity (61%) and strength training (44%) [91]. Dacey et al. (2014) [92] conducted a systematic review to describe curricular components and assess the effectiveness of PA counseling education programs in medical schools [92]. They found four programs with solid evidence of improvements in students’ attitudes, knowledge, and skills in PA counseling, and self-efficacy in conducting PA counseling. Analysis revealed that program duration ranged from 2.5 h to 4 years, indicating that varying lengths and intensities can have an impact. All programs provided opportunities to practice PA counseling, and three had interventions grounded in a conceptual framework [92]. Two programs addressed students’ PA behavior. The four programs with the strongest evidence of impact incorporated PA into existing programs addressing behavioral change or health promotion/disease prevention topics. This suggests support for developing PA counseling skills by integrating them into existing curricula [92]. One study explored medical students’ views on an e-learning resource, MEdic Gaming (MEGA), focusing on PA promotion and prescriptions [93]. The results showed that despite receiving limited instructions on PA prescriptions, medical students recognized the importance of exercise in preventing and managing chronic conditions. The study found that students found the MEGA e-learning resource informative and believed that it could be incorporated into undergraduate medical curricula. However, the students also emphasized the need for supplementary e-learning on PA prescriptions, such as patient counseling opportunities in person, either through dedicated teaching sessions or during clinical placements. It is unclear whether addressing medical students’ deficits in PA knowledge has a positive impact on their future clinical skills [93].

The inclusion of PA training in undergraduate medical education is often inadequate. A study of Australian medical schools revealed that while most institutions (88.2%) provided some form of PA training, the depth and breadth of this training were often insufficient. Only 42.9% of schools believed that their PA training was adequate to prepare students for effective patient counseling [88]. Moreover, only 46.7% of participants included national strength training recommendations in their curricula. This inadequacy is reflected in the fact that many inactive Australians still need to receive PA recommendations from general practitioners [88]. These results indicated that providing PA on an optional basis does not provide the essential knowledge and skills that future physicians must possess. Jadczak et al. (2019) introduced a 1.5 h PA module into a 5th-year geriatric medicine course that included an exercise tutorial and practical counseling session. This intervention significantly improved students’ perceived competence in advising older adults about exercise [94]. Recognizing the need for practical application, a student-led initiative at the University of British Columbia Faculty of Medicine integrated exercise prescriptions into the curriculum, emphasizing practical skills. This approach allowed students to deepen their understanding through real-life case-based sessions, thus providing a hands-on learning experience. The curriculum covered the PA Vital Sign10 assessment, identifying safety for starting exercise, creating personalized exercise prescription plans, and overcoming barriers through motivational interviews or referrals to exercise professionals. The content was integrated into the existing curriculum with the support of curriculum leaders, adapted from existing sessions, and included in case-based sessions on relevant clinical topics. By implementing this approach, students can enhance their abilities and deepen their understanding of the subject matter by applying their knowledge to practical situations that mirror real-life situations [95]. Similarly, a review of PA education in US medical schools found that more than half of the institutions did not offer any formal PA education, leaving many future physicians ill prepared to assist their patients with PA counseling [96]. According to the results, a significant number of institutions did not offer PA education-related courses, and when available, they were rarely mandatory [96]. Courses focused on sports medicine and exercise physiology are more common than those addressing behavioral counseling, lifestyle medicine, and preventive medicine [96]. Weiler et al. (2012) [97] conducted a study evaluating the provision of PA teaching content in the curricula of all medical schools (31) in the UK [97]. The researchers discovered that the delivery of PA teaching varies greatly across UK medical schools, with much of it being either scarce or nonexistent [97]. The increasing prevalence of integrated curricula hinders the incorporation of PA teaching into existing and well-established curricula traditionally based on medical specialties. Additionally, there is a widespread lack of fundamental PA teaching elements in PA education, such as endorsements and guidance from all four UK departments of health [97].

Despite these challenges, there are initiatives aimed at improving PA training in residency programs. For example, a project in the US successfully integrated PA counseling into primary care residency training, showing improved knowledge and practice among residents [98]. One study in Ohio found that family medicine, internal medicine, and obstetrics/gynecology residency programs dedicated an average of 2.8 h per year to didactics on obesity, nutrition, and PA (ONPA) topics [98]. Ten programs taught techniques for health behavior counseling. The results indicated that exposure to ONPA-related didactics was associated with greater knowledge of counseling techniques among residents. However, those with ONPA-related didactics exhibited poorer attitudes and perceived professional norms toward ONPA counseling. Interviews with residents from all three specialties revealed similar barriers to ONPA counseling, but there was variation in the perception of responsibility to provide such counseling. Although primary care physicians are expected to counsel overweight and obese patients, few residency programs offer adequate training to support such counseling [98]. Connaughton et al. [99] investigated US medical school deans and education directors, focusing on PA, exercise topics, and graduating students’ competency in exercise prescription. They found that lack of training was a significant obstacle for physicians in providing PA counseling to patients. Data were collected from 72 of 128 medical schools, resulting in a response rate of 56%. Only 10% of the respondents said that their students could design exercise prescriptions, and only 6% reported that their school provided a course addressing the American College of Sports Medicine Guidelines for Exercise Testing and Prescription [99].

Moreover, integrating PA education into continuous medical education can help address the ongoing educational needs of physicians, who may not have received sufficient training during their formal education. The Exercise is Medicine Education Committee, a collaboration between Exercise is Medicine and the American College of Sports Medicine, has determined that medical students should acquire expertise in four critical domains by the end of their training: performing PA and fitness assessments, prescribing and supporting exercise programs, providing counseling, using behavioral strategies, and promoting their own physical and mental health [100].

It is important to note that despite the long-standing recognition of the importance of PA education and sporadic attempts to address this issue, deficiencies in PA education persist globally in medical school curricula. Studies have consistently revealed the inadequacy of PA training, particularly in terms of exercise prescription skills and behavioral counseling [101,102,103,104,105,106]. To address this need, the American Medical Society for Sports Medicine (AMSSM) has developed an Exercise Medicine and PA promotion (EM-PAP) curriculum. The medical school curriculum includes exercise prescriptions for common medical conditions and facilitates discussions on how diseases can be modified by exercise and PA (Table S2, Supplementary Material) [107]. If incorporated, every physician completing medical school will possess the necessary skills to prescribe PA for common medical conditions. Within residency programs, the promotion of PA and exercise can be incorporated into the existing curriculum, or as part of a broader lifestyle medicine curriculum that encompasses training in promoting PA, healthy nutrition, and proper sleep hygiene. The EM-PAP curriculum within sports medicine fellowship training offers in-depth and focused experiences with clinical encounter benchmarks that may vary based on resources and infrastructure [107]. The curricula provide guidance on promoting PA at various levels of medical education; however, some limitations and variations exist, such as differences in institutional and program approaches to education and residency education variations between specialties [107]. A comprehensive study by Páez et al. (2024) [108] highlighted the effectiveness of the “Exercise is Medicine” (EIM) workshops in Colombia, which trained over 4000 healthcare professionals. The workshops included theoretical and practical components using interactive lectures, hands-on elements, and role-playing. These methods significantly improved participants’ knowledge and awareness of PA prescriptions and provided practical tools for clinical practice. The EIM approach emphasizes individualized PA prescriptions considering patients’ beliefs, motivations, needs, and barriers (Table S2, Supplementary Material) [108]. These initiatives aim to prepare future physicians to effectively counsel patients on PA, thereby improving patient outcomes and addressing the global challenges of NCDs. The varied approaches to assessment and instruction reflect diverse educational contexts and needs but collectively contribute to a robust framework for incorporating exercise medicine into medical education.

## 3. Survey Studies among Medical Students

### 3.1. Perceptions and Knowledge of Nutrition

The perceptions and knowledge of medical students about nutrition are crucial for their future role as healthcare providers. Despite the growing recognition of nutrition as a critical component of health and disease management, many studies have highlighted substantial gaps in medical education regarding nutrition, significantly impacting student preparation to effectively counsel patients [109,110,111,112,113,114,115,116,117,118,119,120,121,122,123,124,125,126] A cross-sectional study in the US showed that only 29% of medical interns felt sufficiently trained in clinical nutrition during medical school, highlighting a significant deficiency in nutritional training. This lack of preparedness was associated with fewer weeks of nutrition education, underscoring the need for more comprehensive curricular coverage [127]. Similarly, a study at the Indiana University School of Medicine found that both medical students and residents perceived current nutrition education as inadequate, often outdated, and poorly integrated into the curriculum (Table S3, Supplementary Material) [128]. In Australia and New Zealand, final-year medical students have expressed a need for more robust nutrition education, feeling unprepared to handle nutrition-related issues in clinical settings. This indicates the need for curricular reforms to include more comprehensive and practical nutrition training (Table S3, Supplementary Material) [129].

Various efforts have been made to enhance nutritional education. At the University of Alabama at Birmingham, a program involving medical students in community cooking demonstrations significantly improved their nutritional knowledge and understanding of social determinants of health. Participants reported improved skills in nutritional counseling and better communication with patients from diverse backgrounds [130]. Similarly, at Florida International University, transitioning from take-home assignments to large-group application exercises in an endocrinology course significantly improved students’ perceptions of their learning about nutrition and social determinants of health [131]. Experiential learning has shown promise in enhancing nutritional education. A study at the University of Cincinnati College of Medicine implemented a medical nutrition therapy experience in which medical students followed medically prescribed diets for two weeks. This immersive approach increased students’ confidence in using nutrition counseling in clinical settings and underscored the importance of practical hands-on experiences in nutrition education [132]. Despite these efforts, many medical schools lack dedicated nutrition courses, often integrating nutrition into other subjects, without providing sufficient depth. This integration often results in graduates feeling unprepared to apply their nutritional knowledge to clinical practice [127]. An international review highlighted that while there is global recognition of the need for better nutrition education, the actual implementation in medical curricula remains inconsistent, leading to significant variability in students’ readiness [133].

From a historical perspective, the fluctuating importance of nutrition in medical education is evident. Advances in medical science and the emergence of new fields have often overshadowed nutrition, reducing its emphasis on curricula. However, innovative approaches such as problem-based learning and case studies have shown potential in improving students’ nutritional knowledge and counseling skills (Table S3, Supplementary Material) [134]. A study at the University for Development Studies in Ghana found that 92% of clinical-level medical students considered nutrition education relevant to their future practice. However, 70% were dissatisfied with the amount of time dedicated to nutrition education. Only 22.2% felt adequately prepared to provide nutritional care, highlighting the need for improvements in both the content and format of nutrition education (Table S3, Supplementary Material) [135]. Further emphasizing these gaps, a survey conducted at Monash University in Australia found that medical students perceived nutrition as a foundational component of medical management, particularly for chronic diseases. However, the emphasis on nutrition varies significantly among educators, affecting students’ perceptions of its importance and their preparedness to integrate it into clinical practice (Table S3, Supplementary Material) [136]. A review of interventions aimed at improving nutrition education for medical students found that, while various strategies, including lectures, workshops, and interactive modules, can enhance knowledge, many students still feel underprepared for clinical applications. This underscores the need for a more integrated and practical approach to nutrition education in medical curricula [137].

Research from Gulf Medical University in the UAE found that 78.5% of medical graduates felt that they had not received adequate nutrition instruction during their undergraduate studies. Graduates have identified major areas of deficit in clinical nutrition, primary care, and evidence-based nutrition, highlighting the need for curricular improvements (Table S3, Supplementary Material) [138]. A recent study by Amoore et al. (2023) [84] in Ghana demonstrated that nutrition education interventions significantly improved medical students’ dietary habits, knowledge, and self-efficacy in providing nutrition care. The study emphasized the need for continuous implementation of such interventions to sustain these outcomes [84]. Another study at the University of Birmingham integrated nutrition education and medical training through community-based culinary workshops. This innovative approach enhances students’ counseling confidence and practical skills, reflecting the importance of experiential learning in nutrition education (Rothman et al. 2020) [139]. The University of Cambridge has also implemented a comprehensive nutrition education initiative utilizing a vertical spiral approach during the years of clinical focus on medical degrees. This program, which includes leadership and advocacy training, diverse teaching modes, and a multidisciplinary approach, has shown promise but continues to require ongoing evaluation and adaptation to fully meet the needs of medical students [140].

In conclusion, medical students globally recognize the critical role of nutrition in patient care but consistently report feeling inadequately prepared to address nutritional issues in clinical settings. Enhancing nutrition education through dedicated courses, practical workshops, and integrated learning approaches is essential to equip future physicians with the necessary knowledge and skills. Addressing these educational deficiencies is crucial for improving public health outcomes and ensuring that future doctors provide optimal nutritional guidance to patients. This consensus has been supported by various studies conducted in different countries.

### 3.2. Perceptions and Knowledge of Physical Activity

Medical students’ perceptions and knowledge regarding PA have been extensively studied, highlighting significant gaps in their education and training. Despite recognizing the importance of PA in health promotion and disease prevention, many medical students feel inadequately prepared to counsel patients effectively [141,142,143]. In the UK, studies have shown that medical students are less knowledgeable about PA guidelines than about other health promotion guidelines, such as those for alcohol consumption. This was evidenced by only 40% of UK medical students being aware of the national PA guidelines, and only 52% feeling adequately trained to advise patients on PA (Table S4, Supplementary Material) [144]. Similarly, a study of American medical students found that although they received more instruction on exercise during their preclinical years, they felt less confident in applying this knowledge during clinical practice, highlighting a disconnect between theoretical learning and practical application (Table S4, Supplementary Material) [145]. Turkish medical students also reported limited knowledge and training in PA promotion, with many expressing a desire for more education on this topic (Table S4, Supplementary Material) [146]. Further research has highlighted that final-year UK medical students were largely unaware of the current exercise guidelines, and many had not received recent training in lifestyle medicine, suggesting a significant need for curriculum reforms to better equip future doctors (Table S4, Supplementary Material) [147]. In the United States, despite a high interest in training related to chronic disease prevention, most students reported minimal formal education in PA and nutrition, underscoring the need for more structured and extensive training programs (Table S4, Supplementary Material) [148]. A study assessing the PA and fitness levels of undergraduate medical students in India found that, while the majority met the recommended PA levels, their physical fitness did not reach the desired level. This indicates that being physically active does not necessarily equate to being physically fit and underscores the need for comprehensive fitness training [149]. Additionally, a PA learning module implemented in New Zealand demonstrated that structured educational interventions could significantly improve medical students’ confidence and perceived competence in providing PA advice [150]. Overall, these studies highlight a clear need for enhanced education and training in PA and lifestyle medicine within medical school curricula. Medical students globally recognize the critical role of PA in health promotion; however, their current training is insufficient to adequately prepare them for clinical practice. Integrating comprehensive PA and lifestyle medicine modules along with practical training opportunities could significantly improve medical students’ confidence and competence in this vital aspect of preventive medicine [151,152,153,154]. Addressing these educational deficiencies is crucial to improving public health. Future physicians must be equipped to provide optimal guidance to their patients, and systematic changes in medical curricula are essential to bridge this gap. Ensuring that medical students receive robust training in PA and lifestyle medicine will enable them to effectively combat the growing prevalence of chronic diseases through informed and confident patient counseling.

## 4. Integrating Lifestyle Medicine into Medical Curricula: The Indissociable Roles of Nutrition and Physical Activity

Nutrition and PA are interrelated in achieving optimal health. By integrating these elements into medical education and practice, practitioners can effectively address the root causes of chronic diseases and improve the long-term health outcomes for their clients. These guidelines serve as a valuable resource for practitioners seeking to enhance their practice through a combined focus on nutrition and exercise, ultimately contributing to better public health [155]. By integrating lifestyle medicine into the curriculum, physicians can be better prepared to address the root causes of these diseases through preventive and therapeutic strategies [156,157]. Furthermore, evidence-based practice guidelines for nutrition and exercise practitioners emphasize the importance of providing individualized and consistent education to improve client outcomes, highlighting the need for comprehensive training that encompasses both nutrition and PA [155]. Interestingly, despite the recognized importance of this integrated approach, studies have found that knowledge among specific populations, such as general practitioners, who are well positioned to guide patients, often demonstrates a greater inclination to advise on PA rather than nutrition, suggesting an imbalance in current medical guidance practices [158]. This underscores the need for a more unified approach to medical education to ensure that healthcare professionals are equally proficient in both areas.

Lifestyle medicine focuses on educating patients about the benefits of a whole-food, plant-predominant dietary lifestyle, regular PA, healthy sleep habits, stress management, avoidance of risky substances, and positive social connections as primary therapeutic modalities for the treatment and reversal of chronic diseases [159]. Traditionally, these principles have been taught under the guise of biochemistry-centric nutrition lectures, recommended guidelines for PA, and selected topics in preventive medicine during preclinical years. However, this approach often fails to provide students with the practical skills required to apply this knowledge in clinical settings [159]. Incorporating lifestyle medicine into medical education can inspire students to choose primary care. Students become more adept at addressing and treating the root causes of chronic diseases, which can reignite their initial desires to treat and heal and may have inspired them to pursue medicine first [159]. This is particularly important given the current shortage of primary care providers and the increasing burden of chronic diseases. Research has shown that the training of healthcare professionals can positively affect individual lifestyles. For example, healthcare professionals are more likely to adopt healthy eating behaviors and responsible drinking habits that they can then pass on to patients [160]. This highlights the importance of incorporating comprehensive lifestyle medicine education into medical curricula to promote healthy behavior among future healthcare providers and patients. Moreover, integrating lifestyle medicine into medical education aligns with broader goals of public health and preventive medicine. This helps physicians understand the complex interplay between lifestyle factors and health outcomes, thereby promoting a more holistic approach to patient care [161]. By addressing lifestyle factors such as diet and PA, medical students can learn to provide more comprehensive and effective patient care.

**Figure 1:**
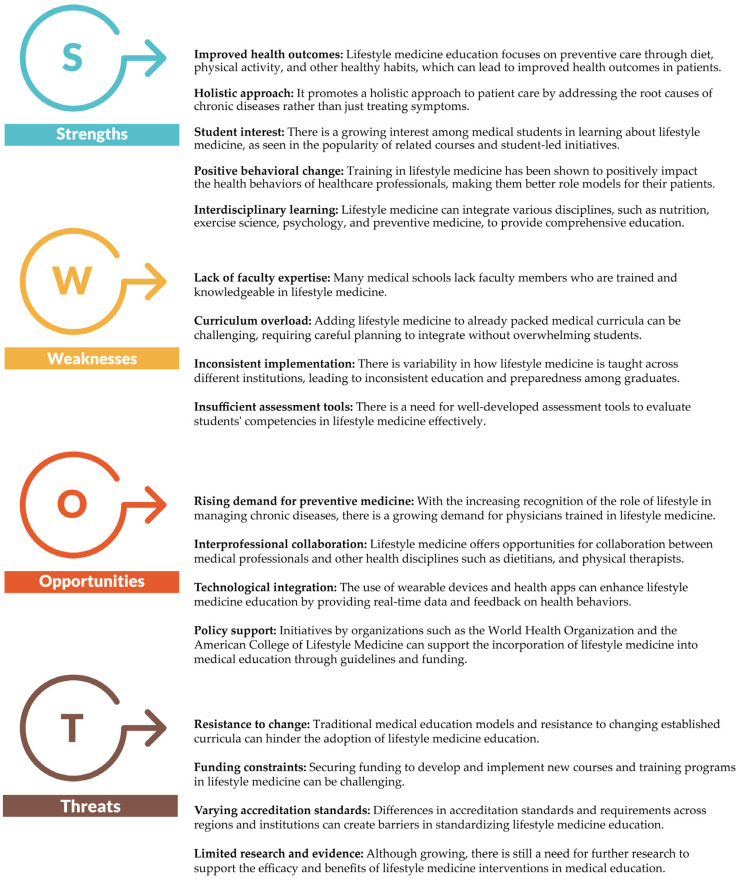
Comprehensive SWOT analysis of integrating lifestyle medicine into medical school curricula, detailing the strengths, weaknesses, opportunities, and threats associated with the implementation and education of lifestyle medicine among medical students.

The implementation of lifestyle medicine in medical education has yielded positive global outcomes. For example, Riphah International University in Pakistan successfully integrated lifestyle medicine into its undergraduate curriculum, which included a training faculty and embedding lifestyle medicine content throughout educational activities [162]. This comprehensive approach ensures that students are well versed in lifestyle medicine principles from the start of their medical education. At Stanford Medical School, a lifestyle medicine course available to all university students has gained popularity, demonstrating growing interest in this field. The course covers a wide range of topics, including nutrition, exercise, sleep, and behavioral change techniques, and is supported by faculty from various disciplines [163]. Such interdisciplinary courses highlight the broad applicability and appeal of lifestyle medicines. The American College of Lifestyle Medicine has been instrumental in developing educational materials and promoting lifestyle medicine education. Their initiatives, such as the Lifestyle Medicine Residency Curriculum and various Continuing Medical Education courses, aim to fill the gaps in current medical education and ensure that physicians are well equipped to incorporate lifestyle medicine into their practice [164]. Figure 1 shows a comprehensive SWOT analysis that outlines the strengths, weaknesses, opportunities, and threats related to incorporating lifestyle medicine education into medical curricula, emphasizing the integration of nutrition and PA.

## 5. Conclusions

Numerous efforts have been made to include nutrition and PA in medical curricula; however, the task has proven challenging owing to various challenges and barriers. The expansive scope of medical education, which includes a wide array of subjects, clinical rotations, and research obligations, leaves limited room for additional curricular and extracurricular components. Time constraints further exacerbate this issue, impacting the amount of content that can be covered and posing challenges in dedicating sufficient time for experiential learning and practical application. Resource availability is another significant hurdle that needs to be overcome. Financial constraints, lack of dedicated faculty, insufficient infrastructure for practical sessions, and outdated educational materials impede the development of robust curricular frameworks and the creation of engaging learning experiences. Faculty expertise plays a crucial role in shaping the success of integrating nutrition and PA into medical education. However, this becomes problematic when faculty members lack specialized knowledge of both nutrition and exercise science, behavioral counseling, or curriculum design tailored to these topics. Faculty development programs are essential for bridging this gap; however, their implementation requires substantial institutional support. The broad spectrum of subjects in medical education creates competition for attention and priorities, and resistance to change poses a formidable barrier. Traditional views on medical education may resist incorporating new elements, seeing them as additional burdens rather than essential components. Overcoming this resistance requires strategic communication, faculty buy-in, and demonstration of the value of nutrition and PA education in producing well-rounded and competent physicians. To summarize, integrating nutrition and PA education into medical curricula presents numerous challenges that demand a comprehensive understanding of institutional dynamics, faculty perspectives, and the intricate interplay between educational priorities. Addressing these challenges is imperative to bring about a transformative era in medical education that emphasizes the vital roles of nutrition and PA in healthcare. Overcoming these challenges necessitates a commitment to educational innovation, the proactive involvement of educators, and a responsive approach to the evolving requirements of medical practice. By implementing effective solutions to these challenges, we can shape the future of medical education to ensure that healthcare professionals are well equipped to integrate both nutrition and PA as vital components in providing comprehensive and effective care.

In summary, this manuscript underscores the importance of integrating lifestyle medicine into medical education, particularly in addressing chronic diseases through nutrition and PA. Despite its recognized value, there is an imbalance in medical guidance, with a stronger focus on PA than on nutrition, highlighting the need for a more unified approach. Further research is needed to explore the long-term impacts of lifestyle medicine education and to develop standardized guidelines for its integration. The diversity of educational systems may affect the generalizability of the findings, and implementation strategies may need adaptation to suit specific institutional contexts. The key points to take away are prioritizing faculty training, dedicating sufficient time within the educational curriculum, and incorporating innovative, experiential learning methods such as problem-based learning and community-based programs. Furthermore, fostering collaboration between medical educators, healthcare policymakers, and public health experts can play a pivotal role in overcoming existing barriers, ensuring that future physicians not only possess knowledge but also exhibit confidence in providing lifestyle counseling. By focusing on these areas, medical education can equip physicians to address the global challenges posed by chronic diseases through comprehensive lifestyle medicine.

## Supplementary Materials

The following supporting information can be downloaded at: https://www.mdpi.com/article/10.3390/nu16162809/s1, Table S1. Comparative analysis of nutrition education programs in medical schools. Table S2. Comparative analysis of physical activity education programs in medical schools. Table S3. Medical students’ perceptions and knowledge of nutrition education. Table S4. Detailed analysis of medical students’ perceptions and knowledge regarding physical activity (PA) education.
