# Supplementary material for: Nutrition and Physical Activity Education in Medical School: A Narrative Review
Source: Nutrients. 2024 Aug 22;16(16):2809. doi: 10.3390/nu16162809 (PMC11357297; doi:10.3390/nu16162809)
Supplement: Supplementary file 1 [file nutrients-16-02809-s001.zip › Table S1.pdf]

Table S1. Comparative analysis of nutrition education programs in medical schools: This table provides an in-depth look at the instructional methods, assessment techniques, learner outcomes, and institutional experiences across various medical schools. It highlights the total hours dedicated to nutrition education, the integration of nutrition into the medical curriculum, and the effectiveness of different educational strategies in improving students' clinical skills and knowledge.

Institution	Total hours	Method of instruction	Method of assessment	Learner outcome	Experience outcome	Experience summary	Reference
University of Arizona College of Medicine	75 hours of required nutrition instruction after implementation (compared to 35 hours at baseline)	The nutrition curriculum was integrated into required courses throughout all four years of undergraduate medical studies, rather than being a single course. This integration involved reviewing course syllabi, attending course presentations to identify nutrition content, meetings with faculty to define nutrition objectives, and revising course materials	Objective Structured Clinical Examination (OSCE): The OSCE was used to evaluate applied nutrition skills through a multistation format involving standardized patients. Specific nutrition items were identified for testing and scoring within the OSCE	Improvement in OSCE Nutrition Scores: There was a significant improvement in nutrition OSCE scores for students who received the integrated nutrition curriculum. Scores increased from 41.7% (± 0.9) before implementation to 50.6% (± 1.1) after implementation	The integrated nutrition curriculum was found to be an effective and time-efficient model for enhancing medical students' clinical nutrition skills. The approach also helped distribute nutrition education throughout the entire curriculum, making it more feasible for schools with limited resources	The integrated nutrition curriculum at the University of Arizona College of Medicine successfully increased the total hours of nutrition education, improved students' clinical performance in nutrition as measured by the OSCE, and positively altered students' perceptions of the adequacy of their nutrition education. This model of curriculum	[67] https://doi.org/10.1093/ajcn/73.6.1107.

						integration is considered beneficial for incorporating multidisciplinary information into medical education, with potential applications for other topics	
Harvard Medical School	28 hours	The course includes a combination of problem-based learning tutorials, simulated cases to teach counseling skills, student-led debates, self-assessment exercises, and lectures. Each weekly session pairs a 45-minute lecture with a 90-minute small-group problem-based tutorial where students	Students' diet and exercise habits, and their self-efficacy in counseling about these behaviors, were assessed using a pre- and post-course confidential 43-item written survey	Improved confidence in diet and exercise counseling	87% of students believed the course made them more aware of their dietary choices, and 72% felt their diet had improved. However, only 18% indicated that their exercise habits had improved as a result of the course	An innovative course improved students' confidence in counseling and their own dietary habits.	[53] Conroy, M.B.; Delichatsios, H.K.; Hafler, J.P.; Rigotti, N.A. Impact of a preventive medicine and nutrition curriculum for medical students. Am. J. Prev. Med. 2004, 27, 77–80.

		discuss clinical cases					
University of Nevada School of Medicine	20 hours	Lectures, small group discussions, CD-ROM based modules, and Web-based strategies. The course includes lectures, small group discussions, case studies, and interactive CD-ROM programs from the Nutrition in Medicine series	The assessment methods include OSCE, evaluations from course coordinators, student course and faculty evaluations, graduation questionnaires, alumni surveys, and peer reviews. Additionally, the students' nutrition subscores on the National Board of Medical Examiners Step 1 and 2 Examinations are also used for assessment	The course has been rated highly by medical students (4.4/5.0), and the number of students reporting inadequate nutrition education has decreased significantly from 68% to 11.5%. Their performance in nutrition-related questions on national examinations is above average	The Special Qualifications in Nutrition program has been successful, graduating 22 students from 1999 to 2005, including medical, health ecology, nursing, and PhD students. The nutrition curriculum's integration has been positively reviewed, reaffirming its importance and uniqueness within the medical school	The review process was proactive and positive, leading to recommendations such as increased use of Web-based learning, better integration with clinical faculties, and the development of new opportunities for nutrition education. The process confirmed the importance of nutrition in the medical curriculum and encouraged further integration and support	[54] St Jeor, S.T.; Krenkel, J.A.; Plodkowski, R.A.; Veach, T.L.; Tolles, R.L.; Kimmel, J.H. Medical nutrition: A comprehensive, school-wide curriculum review. Am. J. Clin. Nutr. 2006, 83, 963S–967S.
Ben-Gurion University of the Negev,	10 hours	A nutritional workshop integrated within	Inventory of the nutrition topics and objectives	Over 90% of students answered most of	Students graded the effectiveness of the training on	The ten-hour nutritional workshops within	[68] https://doi.org

Beer-Sheva, Israel		the clinical experience weeks for second-year medical students. The program included lectures, case discussions, dietary intake interviews, debates, and actual practice in class.	using a multiple-choice questionnaire to evaluate nutritional knowledge. A questionnaire to assess the importance of the issues taught and the quality of the teaching. Students also provided suggestions for future courses.	the knowledge test questions correctly. They showed a high level of interest and found the topics relevant to their future careers. However, the ability to identify patients at nutritional risk had lower correct response rates (67% and 61% in the first and second courses, respectively).	a scale of 1–7, with scores ranging between 3.7 to 5.4 in the first year and 3.4 to 5.7 in the second year. Topics considered practical, like obesity, were rated higher than theoretical topics like the Dietary Reference Intake.	the clinical weeks were well-received by second-year medical students. Using cases relevant to the students' age and encouraging active participation through problem-based learning and interactive sessions enhanced their interest and cooperation. Despite some limitations, the program successfully increased students' nutritional knowledge and engagement.	/10.1080/13576280600938232.
New York University School of Medicine,	Not specified	The survey used a previously validated 111-	Multiple-choice quiz	Average correct score on the knowledge quiz was 66%.	Despite recognizing the importance of nutrition	Interns generally agreed that nutrition assessment	[56] https://doi.org/10.1080/07315

University of Pennsylvania, Harvard Medical School, Harvard Pilgrim Healthcare		item close-ended questionnaire.			counseling, interns lacked confidence and knowledge.<br><br>Interns with prior nutrition education reported more negative attitudes towards physician self-efficacy	should be included in routine primary care visits and felt obligated to discuss nutrition with patients.<br><br>However, they lacked the confidence and knowledge to do so effectively.<br><br>Prior exposure to nutrition education did not significantly improve knowledge or attitudes but rather was associated with more negative attitudes regarding the utility of nutritional counseling	724.2008.10719702
University of Louisville;	University of Louisville:32	Integrated curriculum	Embedding nutrition	Improved knowledge of the	Increased interest and participation	The integration of nutrition	[74] doi:10.1177/01

University of Nevada School of Medicine; Tufts University School of Medicine; University of Colorado School of Medicine; University of North Carolina at Chapel Hill; University of North Carolina at Chapel Hill; University of Wisconsin	hours of lecture for the first two years of medical school; University of Nevada School of Medicine: A schoolwide nutrition program with a required medical nutrition course established in 1985. The exact total hours are not specified, but the curriculum is integrated longitudinally throughout the 4-year undergraduate; Tufts University School of Medicine: Specific hours not mentioned, but nutrition was strengthened in the first-year program and added to third-year clerkships	combining basic science with clinical applications.<br><br>Use of small group discussions, standardized patient educators, mentorship programs, and web-based modules.<br><br>Examples include the Nutrition in Medicine (NIM) series and interactive case discussions	questions into examinations and assigning a separate nutrition grade.<br><br>Continuous assessment through both preclinical and clinical years.<br><br>Testing proficiency in specific clinical skills and knowledge through structured assessments like the United States Medical Licensing Examination (USMLE) Step 1	relationship between nutrition and health.<br><br>Ability to take comprehensive nutrition histories and conduct relevant physical examinations.<br><br>Enhanced skills in clinical practice related to nutrition, including diagnosis and treatment of nutrition-related conditions	in nutrition-related courses and electives.<br><br>Positive feedback from students, residents, and faculty regarding the integrated curriculum and its relevance to clinical practice	education into medical curricula has faced barriers such as lack of trained faculty and resources, but innovative teaching methods and strategic planning have led to successful implementation in several institutions.<br><br>There is a recognized need for ongoing support and development to maintain and enhance nutrition education, involving collaboration with various stakeholders, including professional societies and industry partners	48607110376200

and residency programs medical curriculum; University of Colorado School of Medicine: Specific hours not mentioned, but the curriculum is broad and vertically integrated across preclinical and clinical years, emphasizing active adult learning; University of North Carolina at Chapel Hill: Specific hours not mentioned, but the Nutrition in Medicine (NIM) project covers preclinical and clinical training with flexible, modular web-based delivery; University of

	Wisconsin: Specific hours not mentioned, but a Medical Nutrition Handbook is used, emphasizing a problem-based approach to encourage use during clinical patient care						
Two constituent colleges of Cambridge University, England	2 days (intensive weekend workshops)	The intervention consisted of a combination of lectures, demonstrations, simulations, and interactive practical sessions (small group work), incorporating concepts of problem-based learning (mini-PBL). It included both clinical and public health aspects of	Knowledge, Attitudes, and Practices (KAP) scores were assessed using a questionnaire-based instrument before and after the intervention, as well as at a 3-month follow-up. The questionnaire was construct-validated against key clinical learning outcomes, with items randomized	There were statistically significant improvements in KAP scores immediately after the intervention, which were sustained over 3 months. Specifically, mean differences and 95% CIs after intervention were:<br>Knowledge: 0.86 (0.43 to 1.28)	Ninety-seven percent of participants rated the overall intervention and its delivery as 'very good to excellent', and 94% rated the level of teaching as appropriate. Ninety-nine percent demonstrated recall of one or more of the six key take-home messages.	The Need for Nutrition Education Program (NNEdPro) highlighted the necessity for curricular innovation in clinical health nutrition in medical schools. The educational workshops were well received by students from across 15 medical schools. The	[75] Ray, S.; Udumyan, R.; Rajput-Ray, M.; Thompson, B.; Lodge, K.-M.; Douglas, P.; Sharma, P.; Broughton, R.; Smart, S.; Wilson, R. Evaluation of a novel nutrition education intervention for medical students from

		nutrition, as well as practical care planning sessions using validated nutritional screening methods such as the 'Malnutrition Universal Screening Tool' (MUST).	differently at baseline and post-intervention to minimize recall bias.	Attitude: 1.68 (1.47 to 1.89)<br>Practice: 1.76 (1.11 to 2.40)<br>Overall KAP: 4.28 (3.49 to 5.06)		project established the effectiveness and acceptability of an intensive nutrition education intervention, showing significant improvements in KAP scores. The multidisciplinary approach and innovative teaching methods contributed positively to the outcomes, suggesting a viable model for curricular change in medical education.	across England. BMJ Open 2012, 2, e000417
Harvard University and The Culinary Institute of America	The program is a 4-day conference.	Didactic presentations<br>Interactive (hands-on cooking) plenary	Anonymous survey of registrants' self-reported nutrition-related behaviors at the	Significant positive changes in the frequency of cooking their own meals.	Changes in personal and professional nutrition-related behaviors reported by	The inclusion of culinary education combined with traditional didactic, nutrition	[76] Eisenberg, D.M.; Miller, A.M.; McManus, K.; Burgess, J.;

sessions and workshops<br><br>Culinary education in the form of cooking demonstrations and participatory hands-on cooking workshops<br><br>Nutritional epidemiology and physiology lectures<br><br>Exercise and mindfulness sessions	start of the conference and 12 weeks later.<br><br>Analysis using the χ^2 test for 2×2 tables to test for the equality of proportions.<br><br>Investigation of the relationship between personal nutrition behaviors and professional counseling behaviors with Spearman rank correlations.	Increased personal awareness of calorie consumption.<br><br>Increased frequency of vegetable, nut, and whole grain consumption.<br><br>Improved ability to assess a patient's nutrition status.<br><br>Enhanced ability to successfully advise overweight or obese patients regarding nutritional and lifestyle habits.	participants before and 3 months after the educational experience.	presentations resulted in measurable positive changes in both personal and professional nutrition-related behaviors among participating healthcare professionals.<br><br>Limitations include modest sample size, response rates, the anonymous nature of the survey, and the short follow-up period.<br><br>Further investigation is warranted to study the sustainability of these changes and their impact on patients' behaviors and	Bernstein, A.M. Enhancing medical education to address obesity:"See one. Taste one. Cook one. Teach one.". JAMA Intern Med 2013, 173, 470-472.

Boston University School of Medicine (BUSM)	The curriculum includes over 20 hours of nutrition education during the preclerkship years.	Lectures, case-based learning, clinical rotations, student-mentored projects, virtual curriculum	Written Examination: USMLE step 1 and step 2 nutrition subscores.<br><br>Formative Assessments: Pre- and post-tests, student and staff surveys.<br><br>Summative Assessments: Competency-based evaluations.	Improved knowledge, attitudes, and practice skills in nutrition medicine.<br><br>Increased confidence in providing nutritional counseling to patients.	Development of leadership skills through the Student Nutrition Awareness and Action Council.<br><br>Enhanced interprofessional collaboration between medical students and dietetic interns.<br><br>Participation in regional nutrition- and obesity-related committees and community outreach programs.	clinical outcomes over time.<br><br>The integration of a nutrition curriculum at BUSM, guided by a Vertical Integration Group, involved both classroom and clinical education methods supplemented by extracurricular activities. These efforts aimed to improve the overall competency in nutrition medicine among medical students. The program's success was marked by enhanced student leadership, interdisciplinary collaboration, and	[77] Lenders, C.; Gorman, K.; Milch, H.; Decker, A.; Harvey, N.; Stanfield, L.; Lim-Miller, A.; Salge-Blake, J.; Judd, L.; Levine, S. A novel nutrition medicine education model: the Boston University experience. Adv Nutr 2013, 4, 1-7.

						positive feedback from students and faculty. Future includes further evaluation and dissemination of the model to other institutions	
University of Cambridge, School of Clinical Medicine	Undergraduate Degree (6 years)<br>Clinical Year 1: 4-hour session<br>Clinical Year 3: 2-hour session<br>Graduate Degree (4 years)<br>Clinical Year 1: 1.5-hour session	Undergraduate Degree<br>Clinical Year 1: Plenary session followed by three "carousel-style" workshops facilitated by a multidisciplinary team (doctors, dietitians, nutritionists, nurses)<br>Clinical Year 3: 2-hour session focusing on diet-disease relationship and lifestyle behavior<br>Graduate Degree	Pre- and post-teaching multiple-choice questionnaire measuring nutrition knowledge, attitudes, and practices.<br>Qualitative feedback through written evaluations from students and peer-facilitated feedback for workshop facilitators.	Increased nutrition knowledge and improved attitudes towards the importance of nutrition in clinical practice.<br>Example: 70% of students strongly agreed on the importance of nutrition in reducing global disease burden post-session (from 0% pre-session).	Positive feedback on the comprehensiveness and interactivity of workshops.<br>Suggestions for more teaching time and inclusion of additional nutritional topics.	The initiative at the University of Cambridge aimed to enhance medical nutrition education through a structured curriculum involving both undergraduate and graduate students. It employed a variety of teaching methods, including interactive workshops and sessions facilitated by multidisciplinary	[140] Ball, L.; Crowley, J.; Laur, C.; Rajput-Ray, M.; Gillam, S.; Ray, S. Nutrition in medical education: Reflections from an initiative at the University of Cambridge. J. Multidiscip. Healthc. 2014, 7, 209–215.

		Clinical Year 1: Similar session to undergraduates but condensed to 1.5 hours due to smaller cohort size				teams. The approach showed promising improvements in student knowledge and attitudes, though continuous evaluation and enhancement were necessary to optimize the program.	
Boston University School of Medicine	Preclinical curriculum: Approximately 27 hours<br>Clinical curriculum: 12 hours<br>Total: 39 hours	Extracurricular activities (e.g., Supplemental Nutrition Assistance Program (SNAP) challenge, Test Kitchen)<br>Mandatory online modules<br>Formal didactics<br>In-class practice of basic nutrition and behavioral health counseling	Electronic surveys to assess knowledge and confidence<br>Participation in events and activities<br>Continuous curriculum review and feedback from course directors	Increased knowledge, attitude, and skills in medical nutrition<br>Improved understanding of nutrition resources and skills<br>Enhanced ability to counsel patients regarding healthy lifestyles	Students reported gaining a better understanding of the role dietitians play in healthcare<br>Increased empathy and understanding of challenges faced by underserved populations<br>Improved skills in motivational interviewing and dietary history taking	The Student Nutrition Awareness and Action Council (SNAAC) at Boston University School of Medicine was created to address the lack of nutrition education in medical training. Through a multifaceted approach, including	[78] Schoettler, C.L.; Lee, J.N.; Ireland, K.A.; Lenders, C.M. A Novel Method of Increasing Medical Student Nutrition Awareness and Education. J Biomed Edu 2015, 2015, 1-8,

Service-learning projects		extracurricular activities, formal modules, and service-learning projects, SNAAC aims to improve medical student knowledge and skills in nutrition. The initiative includes various events like the SNAP challenge and Test Kitchen to provide practical experiences. The program has led to increased student involvement, better understanding of nutrition's role in healthcare, and the integration of more nutrition education into the medical school curriculum	doi:10.1155/2015/784042.

Goldring Center for Culinary Medicine, Tulane University School of Medicine, New Orleans	The elective is a 28-hour course comprising an eight-module series	The program features premodule 30-minute videos on lecture content with premodule quizzes.<br>45 minutes of precooking classroom discussion on gaps in knowledge shown in quiz responses.<br>1.5 hours of hands-on cooking to illustrate clinical and pathophysiology points from the lecture material.<br>45 minutes for eating the prepared meal during discussion of national board-style questions drawn from the module material	A 59-question panel survey was conducted twice annually from September 2012 to May 2014.<br>Surveys included questions on dietary habits, attitudes, and competencies (DACs) regarding nutrition counseling for patients<br>Responses were collected using three-point Likert scales for attitudes and competencies and a six-point scale for dietary habits, later translated to a three-point scale for analysis	Simulation-based medical education with deliberate practice (SBME-DP) versus traditional education significantly improved fruit and vegetable diet (OR = 1.38, 95% CI: 1.07–1.79, p = 0.013).<br>Improved attitudes towards the importance and efficacy of nutrition counseling (OR = 1.81, 95% CI: 1.40–2.35, p < 0.001).<br>Enhanced competencies in providing nutrition counseling (OR = 1.72, 95% CI: 1.54–1.92, p < 0.001)	The elective significantly increased the odds of students reporting positive attitudes about the importance and efficacy of nutrition counseling.<br>Students who participated in the elective showed significant improvements in their own dietary habits, specifically in daily intake of fruits and vegetables	The hands-on cooking and nutrition education elective at Tulane University's Goldring Center for Culinary Medicine was superior to traditional clinical education.<br>The program included a comprehensive curriculum combining theoretical knowledge with practical cooking experience, aiming to improve medical students' competencies in nutrition counseling.<br>The study demonstrated significant	[79] Monlezun, D.J.; Leong, B.; Joo, E.; Birkhead, A.G.; Sarris, L.; Harlan, T.S. Novel longitudinal and propensity score matched analysis of hands-on cooking and nutrition education versus traditional clinical education among 627 medical students. Adv Prev Med 2015, 2015, 656780.

						improvements in students' dietary habits, attitudes towards nutrition counseling, and their competencies in providing nutrition advice to patients	
Geisel School of Medicine at Dartmouth	While the National Research Council recommends a minimum of 25-50 hours of nutrition education, the exact total hours for the Geisel curriculum are not explicitly stated but encompass multiple courses and clerkships throughout the 4-year curriculum	The curriculum utilizes a variety of instructional methods, including traditional lectures, practical experience in motivational interviewing and nutrition counseling, and case study discussions. Content is also integrated into courses on metabolism, microbiology, gastroenterology,	The curriculum includes assessment methods such as performance of nutrition assessments, accurate measurement of anthropometrics, nutritional exams to assess malnutrition, and the development and implementation of nutrition plans. Additionally, the curriculum mapping allows	The primary learner outcome is to empower medical students with comprehensive nutrition knowledge, enabling them to work collaboratively with registered dietitians and other healthcare team members to support patient care. Specific competencies include understanding	The curriculum aims to enhance students' practical skills in nutrition assessment and counseling, promote positive behavioral changes through motivational interviewing, and translate nutrition science into practical information for patients, families, and peers. Additionally, it focuses on	The experience summary includes the development and integration of a formalized nutrition program, the Nutrition in Medical Education Program, which is embedded throughout the 4-year curriculum. The program involves a combination of lectures, practical experiences, and	[80] doi:10.1007/s40670-018-00629-6

		family medicine, obstetrics and gynecology, rheumatology, and orthopedics	for the identification of gaps and consistent implementation of objectives	core nutrition science concepts, applying nutrition knowledge to manage health and disease, and promoting nutrition in clinical and public health settings	fostering collaboration and teamwork in nutritional practice	community-based opportunities. The curriculum is continuously evaluated and adapted to ensure comprehensive nutrition education	
University of Alabama at Birmingham School of Medicine (UAB SOM)	Training session: 3 hours<br>Farmer's market cooking demonstration: 2 hours<br>Optional debriefing session: 1 hour<br>Total per session: 6 hours	Social determinants of health education<br>Motivational interviewing<br>Disease-based nutrition science<br>Food insecurity awareness<br>Hands-on cooking lesson	Post-experience surveys using Likert-type rating scale<br>Free-text response questions<br>Analysis of recurring themes using a modified grounded theory approach	Increased knowledge in nutrition education and social determinants of health<br>Improved communication skills<br>Enhanced ability to counsel patients on nutrition	High engagement and positive reception from students<br>Development of practical skills in a community setting<br>Recognition of the importance of social determinants of health in patient education	The CHOP (Cooking Healthily on a Penny) program is a community-engaged learning initiative at the University of Alabama at Birmingham. Medical students participate in weekly cooking demonstrations at a local farmer's market, after receiving comprehensive training. This experience	[130] https://doi.org/10.4103/efh.EfH_234_19

includes a 3-hour training session, a 2-hour market demonstration, and an optional 1-hour debriefing. The training covers essential topics such as nutrition science, social determinants of health, and motivational interviewing. The program aims to equip future physicians with practical skills for nutrition counseling while understanding the social factors affecting health. Over three years, 117 students participated, with a high satisfaction rate and significant reported learning

						outcomes in nutrition education and patient interaction (cooking demonstrations_ …)	
Donald and Barbara Zucker School of Medicine at Hofstra/Northwell, Hempstead, New York, USA	The workshop was integrated into a 9-week course titled “Fueling the Body.” The specific workshop session dedicated to clinical nutrition lasted for 2 hours	The workshop followed a flipped-classroom model, where students completed pre-session readings and a three-day food diary. On the workshop day, there was a 20-minute interdisciplinary framing discussion, followed by small group discussions with clinical case vignettes. The session was co-facilitated by physicians and	The students' performance was assessed using a pre- and post-session online survey, as well as an OSCE at the end of the course. The OSCE included checklist items completed by standardized patients to evaluate the students' nutritional assessment skills	Post-workshop, 70% of students felt they had sufficient knowledge to counsel patients on nutrition (up from 38%), 68% felt comfortable completing a nutritional assessment (up from 35%), and 63% felt confident advising patients about nutrition (up from 32%). Students in the workshop outperformed a control cohort on the nutrition-related	The workshop led to a significant increase in students’ self-assessed knowledge, comfort, and confidence in providing nutritional care. It also resulted in improved performance in OSCE nutrition components compared to the control group	The interprofessional nutrition workshop effectively integrated clinical nutrition education into the first-year medical school curriculum. It improved students' confidence and skills in nutritional assessment and counseling. Collaboration between physicians and dietitians provided a robust	[81] doi:10.1080/07315724.2020.1737985

		registered dietitians		component of the OSCE		learning experience and demonstrated the value of interdisciplinary education in medical training	
Herbert Wertheim College of Medicine, Florida International University	The paper does not explicitly state the total hours dedicated to the course or the session. However, it describes a single session conducted within the Endocrinology course.	Initially, the course used a take-home module for second-year students, which included:<br>Background reading<br>Case-based activities for review<br>Written feedback on the case<br>A short literature review	End-of-course surveys for both cohorts<br>The large-group application exercise constituted 1% of the total grade for the Endocrinology course, where students received full credit for attendance and participation in discussion.	Enhanced literature search skills<br>Better understanding of nutritional recommendations for type II diabetes<br>Improved understanding of how social determinants of health affect patient care and health outcomes	The large-group application exercise format led to significantly improved student perceptions of learning compared to the take-home assignment. Students were more likely to agree that the activity improved their skills and understanding of the topics.	The transition from a take-home assignment to a large-group application exercise with minimal faculty facilitation showed:<br>Higher student engagement<br>Improved perceptions of learning outcomes<br>Effective use of resources with minimal faculty involvement	[131] doi:10.1007/s40670-021-01342-7

		Selection of an article for patient education<br>For the subsequent cohort, the method was changed to a large-group application exercise, which included:<br>An introductory lecture<br>Two self-facilitated small-group case-based activities				Potential applicability to other courses and modules with similar resource constraints<br>This format helped students appreciate evidence-based medicine, identify appropriate resources for clinical questions, and provide practical nutritional recommendations considering social determinants of health.	
Donald and Barbara Zucker School of Medicine at Hofstra/Northwell	Workshop 1: 45 minutes large group session + 55 minutes small group session = 100 minutes	Workshop 1:<br>Large group session on nutrition basics led by a registered dietitian and a physician.	Surveys conducted through Qualtrics before the first workshop, after the first workshop, and	Significant improvement in students' self-assessed knowledge, comfort, and confidence in nutritional assessment and	Students reported an increase in their perceived ability to counsel patients on nutrition.<br>The workshops were effective in	The implementation of clinical nutrition workshops significantly improved first-year medical students'	[82] doi:10.3390/nu13114081

Workshop 2: 70 minutes interactive session	Small group sessions led by registered dietitians and physicians, focusing on role-playing nutrition assessments with case studies.<br><br>Workshop 2:<br><br>Interactive large group session conducted virtually via Zoom due to the COVID-19 pandemic.<br><br>Led by a cardiologist and a nephrologist, involving discussions and role-playing dietary counseling using the Ask, Respond, Tell (ART) framework and	after the second workshop.<br><br>Evaluation based on students' self-assessed perceptions using a 5-point Likert scale.	counseling after the workshops.<br><br>Continued high levels of knowledge, comfort, and confidence reported five months after the second workshop.	enhancing students' knowledge, comfort, and confidence in nutritional assessment and dietary counseling.	perceptions of their knowledge and skills related to nutritional assessment and counseling.<br><br>The study supports the integration of clinical nutrition education into the pre-clerkship medical school curriculum to better prepare future physicians for incorporating nutrition counseling into their practice.

		the teach-back method.					
Bar Ilan University Azrieli Faculty of Medicine	Students received a total of 58 hours of lifestyle medicine (LM) teaching, with 49 hours in the pre-clinical years and 9 hours in the clinical years	Frontal Lectures<br>Experiential Learning (e.g., workshops on healthy cooking, yoga, and exercise sessions)<br>Small Group Discussions<br>Role Play with actors<br>Case-Based Learning	Online questionnaires to ascertain students' attitudes, competence, and confidence<br>Semi-structured interviews with course coordinators and department heads	Students' attitudes and confidence in LM counselling improved, with general agreement on the importance of LM and the role of physicians in lifestyle guidance.<br>Specific confidence in areas such as exercise, nutrition, stress management, sleep, and sexuality counseling were measured, showing varying levels of self-perceived confidence.	Staff recognized the importance of LM but cited time constraints and the need for external experts as significant challenges.<br>Students highlighted the need for more practical tools and opportunities for experiential learning	The study concluded that while there was substantial teaching of LM in the pre-clinical years, there was a significant gap during the clinical years. Both students and staff saw the value in LM education, but practical implementation faced challenges such as time constraints and lack of integration into the clinical curriculum	[161] https://doi.org/10.1186/s12909-022-03929-z

VA Boston Healthcare System	4-5 weeks per rotation	The curriculum included various learning activities such as exercise prescription, culinary medicine, and health coaching.<br><br>Instruction primarily occurred during the didactic year	Pre- and post-curriculum surveys, qualitative analysis of clinical vignettes	There was a statistically significant gain in self-efficacy and knowledge of LM competencies among physician assistant students.<br><br>Students demonstrated a shift towards a more patient-centered approach, using motivational interviewing and goal setting	Mixed methods analysis showed a significant improvement in students' clinical language and approach to patient care.<br><br>Pre-curriculum responses were more directive and authoritative, while post-curriculum responses illustrated collaboration and motivational interviewing techniques	The hole Health/lifestyle<br><br>medicine (WH-LM) curriculum effectively improved physician assistant students' abilities to provide holistic, patient-centered care. This was demonstrated through qualitative analysis of clinical vignettes, showing enhanced patient engagement and personalized care strategies	[83] doi:10.1007/s40670-021-01460-2.
University of Cincinnati College of Medicine	The total estimated hours of instruction were 4-6 hours of didactic presentations	Experiential learning through attempting a 2-week medically prescribed diet.<br><br>Participation in small group lunch	Surveys: Nutrition in Patient Care Survey and custom surveys.	Increased confidence in using nutrition counseling in a clinical setting.<br><br>Improved attitudes toward	Overall positive sentiment towards the medical<br><br>nutrition therapy (MNT) experience.	The study demonstrates the educational value of a short, immersive, extracurricular	[132] https://doi.org/10.1007/s40670-023-01771-6

		discussions related to their diet experience.<br>Providing information packets and patient scenarios for clinical context	Qualitative analysis of lunch discussions.<br>Sentiment analysis using NVivo software.<br>The Jefferson Scale of Empathy (JSE) was also used to measure empathy	the physician-patient relationship and physician efficacy.<br>No significant increase in empathy measured by JSE	Varied diet adherence with a majority finding the diet somewhat difficult to adhere to	opportunity in nutrition therapy.<br>Students reported higher confidence and improved attitudes towards the physician-patient relationship and physician efficacy.<br>Recognition of the limitations of routine care and the importance of interprofessional healthcare teams.<br>The MNT experience did not impact empathy as anticipated but provided valuable reflections on interprofessional education	
University for Development Studies,	24 hours	The intervention included multiple teaching and learning activities	Assessments were conducted pre-intervention, post-intervention, and	Nutrition care knowledge: Scores increased significantly from	Positive outcomes: The intervention improved	The nutrition education intervention was effective in	[84] Amoore, B.Y.; Gaa, P.K.; Amalba, A.; Mogre, V.

School of Medicine, Tamale, Ghana		such as interactive lecture presentations using PowerPoint, demonstrations, problem-based learning tutorials, tasting sessions, nutrition games, and role plays.	at 4 weeks follow-up. Data was collected using paper-based questionnaires, measuring lifestyle and dietary habits, nutrition care knowledge, attitude toward nutrition care, and self-efficacy in the provision of nutrition care.	19.49 at baseline to 24.78 post-intervention and slightly decreased to 22.76 at 4 weeks follow-up.<br>Attitude toward nutrition care: No significant change in attitude scores was observed.<br>Self-efficacy: Improved significantly from 5.30 at baseline to 7.02 post-intervention and slightly decreased to 6.44 at 4 weeks follow-up.<br>Lifestyle and dietary habits: Significant improvement in vegetable consumption and moderate-to-vigorous physical	students' nutrition care knowledge, self-efficacy in providing nutrition care, and their own dietary habits and physical activity levels.<br>Sustainability: Continuous implementation is needed to sustain these outcomes.	improving the dietary habits and nutrition care competencies of medical students. Despite slight declines in some outcomes at the 4-week follow-up, the scores remained higher than baseline values, indicating a lasting impact. The study suggests integrating such interventions throughout the medical curriculum to reinforce and sustain these competencies	Nutrition education intervention improves medical students' dietary habits and their competency and self-efficacy in providing nutrition care: A pre, post and follow-up quasi-experimental study. Front Nutr 2023, 10, 1063316.

				activity was observed.			
Philadelphia College of Osteopathic Medicine (PCOM)	The course consisted of four modules, each conducted once in the spring of the first year and once in the fall of the second year for each class of students. Specific total hours are not mentioned directly in the paper.	Two methods of instruction were used:<br><br>Virtual instruction through Blackboard Collaborate, involving cooking demonstrations, lectures, and discussions.<br><br>In-person instruction on campus with hands-on cooking sessions and lectures.	Quizzes on Culinarymedicine .org after reading the required assignments and published articles.<br><br>Completion of four modules: Introduction to Culinary Medicine, Hypertension, Cancer, and Diet and Inflammation.<br><br>Completion of a course survey for course credit.	Knowledge gained (measured through five survey questions with a maximum score of 25 points).<br><br>Enjoyment of the course (measured through two survey questions with a maximum score of 10 points).	Students in the in-person instruction group reported higher levels of course enjoyment compared to the virtual instruction group.<br><br>There was no statistically significant difference in the knowledge gained between in-person and virtual instruction methods.<br><br>The overall student satisfaction with the course was high, regardless of the instruction method, but in-	The study found that culinary medicine education is effective in enhancing medical students' knowledge and enjoyment related to nutrition and cooking skills. While both virtual and in-person instruction methods were effective in knowledge transfer, in-person classes provided a more enjoyable experience. The COVID-19 pandemic's impact on virtual learning posed challenges but did not significantly affect the overall	[85] Glickman, O.; Kakaty-Monzo, J.; Roberts, M.; Daghigh, F. Exploring the effectiveness of virtual and in-person instruction in culinary medicine: a survey-based study. BMC Med Educ 2024, 24, 276.

				person instruction was favored for enjoyment.	knowledge gained by the students
