# Supplementary material for: Nutrition and Physical Activity Education in Medical School: A Narrative Review
Source: Nutrients. 2024 Aug 22;16(16):2809. doi: 10.3390/nu16162809 (PMC11357297; doi:10.3390/nu16162809)
Supplement: Supplementary file 1 [file nutrients-16-02809-s001.zip › Table S2.pdf]

Table S2. Comparative analysis of physical activity education programs in medical schools: This table offers a detailed examination of the instructional methods, assessment techniques, and outcome measurements used in physical activity education programs across different medical institutions. It covers various approaches to teaching exercise physiology, practical experiences, and the overall impact on student competencies and perceptions.

Institution	Total hours	Method of instruction	Method of assessment	Learner outcome	Experience outcome	Experience summary	Reference
Various Canadian medical schools	Exercise physiology is covered as a separate topic in half of the universities, typically with one or two hours of lectures. One university provides six to eight hours of lectures or seminars specifically related to respiratory and cardiac exercise physiology.	Lectures and seminars on exercise physiology and related topics. Practical experience in exercise testing of patients, though provided in less than 40% of colleges. Exercise electrocardiography is taught in just over half of the medical schools.	Not specified	The document suggests that all graduating physicians should have had adequate learning experiences in: Exercise physiology Exercise testing of patients Prevention and care of athletic injuries	Practical experience in exercise testing of patients with respiratory and cardiac diseases. Practical experience in exercise electrocardiography.	Structured and practical learning experiences in sports medicine. Integration of exercise physiology into the medical curriculum. Specific topics recommended for inclusion in the curriculum include exercise physiology, practical exercise testing, physical fitness and conditioning, prevention of sports injuries, emergency treatment of sports injuries, medical services for athletes, and medical rehabilitation. The author suggests that the current curriculum in Canadian medical schools provides limited coverage of sports medicine topics and lacks practical training opportunities in key areas of sports medicine	[101] Cumming, G. Teaching of sports medicine in Canadian medical colleges. CMAJ 1972, 107, 729.
University of Calgary	The length of the sports medicine elective varied by institution. One school offered a 2-week program, most	Instruction methods included sports medicine electives coordinated by university-affiliated units, independent sports medicine facilities, or a combination of both. Teaching was provided by primary care physicians, orthopedic	Not specified	Few family medicine residents in Canada receive clinical training in sports medicine, though most have attended seminars on the subject. The paper highlights a general	The experience outcomes were not explicitly detailed in terms of measurable results. However, it is implied that the limited clinical exposure affects the residents' confidence and competence in dealing with sports medicine issues	The study found that sports medicine is becoming an integral part of family physicians' practice in Canada, yet there is insufficient training in sports medicine during residency. Although many programs offer seminars, very few provide	[102] Wiley, J.; Strother, R.; Lockyer, J. Sports medicine electives. Are they available in Canadian family medicine programs? Can Fam Physician 1993, 39, 1742.

	(10 of 14) offered a 4-week experience, and three schools offered horizontal experiences (details not specified)	surgeons, physiotherapists, and rheumatologists		discomfort among residents in counseling patients about sports-related conditions due to limited encounters with such cases during their training		substantial clinical training in sports medicine. The training that does exist is primarily delivered by primary care physicians, orthopedic surgeons, physiotherapists, and rheumatologists	
Various medical schools in the United Kingdom and Ireland	Not specified	Lectures: Available in different years of medical education across various institutions.<br>Study modules: These allow students to undertake a period of study in sports and exercise medicine, often involving research or in-depth clinical study.<br>Clinical attachments: Students are based in a department where they gain clinical experience and teaching in sports and exercise medicine is coordinated by that department	Multiple-choice questions (MCQ): Used by 4 medical schools.<br>Objective Structured Clinical Examination (OSCE): Used by 4 medical schools.<br>Written exams: Used by 2 medical schools.<br>Written projects/coursework: Used by 1 medical school.<br>Case presentations: Used by 1 medical school.<br>No formal assessment:	The document does not explicitly detail specific learner outcomes but suggests that students gain valuable learning opportunities and that there is a general desire for more exposure to sports and exercise medicine among medical students	The experience outcomes are implied to be positive, with students benefiting from various teaching methods and gaining valuable knowledge and skills in sports and exercise medicine	The document highlights that sports and exercise medicine is being taught formally or informally in many medical schools and there is optimism about the future inclusion of this discipline in undergraduate education. The variety of teaching methods and assessments suggests a comprehensive approach to education in this field	[103] Cullen, M.; McNally, O.; Neill, S.; Macauley, D. Sport and exercise medicine in undergraduate medical schools in the United Kingdom and Ireland. Br J Sports Med 2000, 34, 244-245.

			Indicated by 1 medical school				
University of South Carolina School of Medicine Greenville (USC SOM Greenville)	Not specified	The "Exercise is Medicine" curriculum is integrated into all modules of the undergraduate medical curriculum. This includes Structure and Function, Mind Brain and Behaviour, Medicine and Society, and Clinical Diagnosis and Reasoning. The method of instruction emphasizes active, student-centred, and integrated learning, allowing students to understand the physiological mechanisms, improve communication skills, and learn to guide patients along the behaviour change continuum of exercise adoption and maintenance	Not specified	Medical students are expected to demonstrate proficiency in several areas upon graduation, including Physical Activity (PA) and Fitness Assessment, Exercise Prescription and Implementation, Exercise Counselling and Behavioural Strategies, and Physician's Personal Health	Students gain hands-on experience and develop skills that allow them to effectively counsel patients on exercise and physical activity, improving both their own health and the health of their future patients. The program also includes participation in extracurricular activities like running, cycling, yoga, soccer, and organic gardening to model and encourage healthy behaviours among medical students	The "Exercise is Medicine" curriculum at USC SOM Greenville is a comprehensive and integrated approach to medical education that focuses on the importance of physical activity for disease prevention and treatment. It includes active learning, community partnerships, and extracurricular activities to ensure that future physicians are well-equipped to counsel patients on exercise and improve overall health outcomes	[104] Trilk, J.L.; Phillips, E.M. Incorporating 'Exercise is Medicine'into the university of South Carolina school of medicine Greenville and Greenville health system. 2014, 48, 165-167.
Nottingham University Medical School	The learning module includes a 10-hour referenced slide set series covering key aspects of exercise medicine, chronic diseases, and surgical care	Learning Module: Adapted from an existing and accredited continuous professional development (CPD) training resource.<br>Slide Set Series: A 10-hour referenced slide set series adapted for use from King's College Medical School's undergraduate course	Not specified	Competence in Exercise Medicine: Learners will be equipped to provide specific, effective physical activity advice as part of the prevention, treatment, rehabilitation, recovery, and survivorship of chronic diseases or non-communicable diseases (NCDs).	Proactive Prevention: Doctors will be proactive in disease prevention and specific in their treatment plans with physical activity advice.<br>Influence on Patient Behavior: Trained doctors will be important influencers of patient behavior and key initiators of NCD prevention actions within healthcare systems	Comprehensive Education: The module provides a comprehensive package of teaching resources for undergraduate medical education, ensuring future doctors can integrate exercise prescriptions into their patient treatment options.<br>Availability of Resources: The resources and materials are available to all UK medical schools, offering a base for medical schools to enhance and contribute to the curriculum from their	[105] Gates, A.B. Training tomorrow's doctors, in exercise medicine, for tomorrow's patients. Br J Sports Med 2015, 49, 207-208, doi:10.1136/bjsports-2014-094442.

				Confidence and Capability: Future doctors will be confident, competent, and capable of safe and effective physical activity advice in every consultation		respective departments and healthcare organizations	
University of Otago, Active Living Laboratory, School of Physical Education, Sport and Exercise Sciences, Dunedin, New Zealand	Not specified	The PA Learning Module consisted of three tutorials and one lecture related to PA advising and experiential learning through providing health checks to local residents.	Baseline and post-intervention surveys (paper questionnaires) Awareness of current PA guidelines, benefits of PA, attitudes toward PA advising, perceived competence/importance of PA advising skills, and personal PA habits were assessed using Likert scales.	Increased awareness of PA guidelines and national PA initiatives Improved confidence and perceived competence in providing PA advice	Students reported higher levels of confidence in advising patients about PA Increased appreciation of the importance of PA advising in general practice and the impact of PA on quality of life	The module increased medical students' knowledge of PA guidelines and their confidence in providing PA advice. Even after the intervention, students perceived themselves as only moderately competent in providing PA advice. Suggested that more extensive training in PA advising may be necessary.	[150] Mandic, S.; Wilson, H.; Clark-Grill, M.; O'Neill, D.; Mandic, S. A physical activity learning module improves medical students' skills and confidence for advising patients about physical activity. *Montenegrin J. Sports Sci. Med.* **2018**, *7*, 31–38 [106].
Tehran University of Medical Sciences	2-week elective course	The course employed a combination of lectures, study groups, and practical training. The students attended related clinics with their Sports and Exercise Medicine (SEM) professors to better grasp the	Multiple choice questions (MCQs) Essay questions	The students' knowledge increased significantly in all the topics taught. Most students reported high levels of satisfaction with the	The students showed high levels of satisfaction with the educational materials, and the elective course promoted their knowledge scores in different SEM topics.	The elective course in SEM was offered by TUMS to address the high prevalence of non-communicable diseases (NCDs) and the low level of physical activity in Iran. The course aimed to equip medical	Noormohammadpour, P.; Halabchi, F.; Mazaheri, R.; Mansournia, M.A.; Alizadeh, Z.; Barghi, T.S.; Abolhasani, M.;

		course material through practical training and practice.	Patient management problems<br>Oral examinations<br>Attendance in classes	topics, and there was a significant correlation between their level of satisfaction and their level of knowledge after the course.		students with the necessary skills to prescribe exercise and physical activity for health promotion and NCD prevention. The course followed Harden's 10 steps for curriculum development and included various teaching and assessment methods. The students' knowledge and satisfaction levels increased significantly, indicating the effectiveness of the course.	Kordi, R. Designing and implementing a curriculum for Sports and Exercise Medicine elective course for undergraduate medical students of Tehran University of Medical Sciences. Br J Sports Med 2019, 53, 601-604.
Medical Schools in the USA	Average of 8 hours over 4 years	Foundations of Exercise Medicine and PA Promotion<br>Basics of exercise in health and medicine<br>Basics of exercise prescription<br>Basics of behavioral change<br>Exercise Prescriptions in Medical Conditions and Special Populations<br>Cardiovascular, pulmonary, gastrointestinal, renal, neurosciences, musculoskeletal, endocrine, reproductive, and hematology-oncology conditions<br>Exercise Medicine and PA Promotion in Clinical Practice<br>Writing exercise prescriptions during patient encounters<br>Promoting individual and community engagement<br>Method of assessment: Standardized checklist (e.g., OSCE) for observing student	Understanding the role of exercise in disease prevention/treatment<br>Ability to write general exercise prescriptions<br>Knowledge of behavior change models and practical motivational interviewing	Practical elements include writing exercise prescriptions for patients during clinical rotations and engaging with community resources	Enhanced medical education enabling better integration of exercise medicine in clinical practice	The curriculum emphasizes a combination of theoretical knowledge and practical experience, aiming to prepare medical students to incorporate physical activity promotion into clinical practice	[107] https://doi.org/10.1136/bjsports-2021-104819.

counseling and reviewing exercise prescriptions<br>Short answer or essay exams<br>Written case responses<br>Program evaluation with trainee suggestions
