# Supplementary material for: Nutrition and Physical Activity Education in Medical School: A Narrative Review
Source: Nutrients. 2024 Aug 22;16(16):2809. doi: 10.3390/nu16162809 (PMC11357297; doi:10.3390/nu16162809)
Supplement: Supplementary file 1 [file nutrients-16-02809-s001.zip › Table S3.pdf]

Table S3. Medical students' perceptions and knowledge of nutrition education: This table presents an overview of multiple studies on medical students' perceptions and knowledge regarding nutrition education. It includes data on study types, target sizes, response rates, collection methods, key findings, and the strengths and limitations of each study, providing a comprehensive view of the current state of nutrition education in medical schools.

Study type	Target size/Responses rate	Collection Method	Key Findings	Strengths/Limitations	Reference
Cross-sectional	46 final year medical students / 76%	Questionnaires	71% of the students were interested in nutrition, and 94% believed it should be included in the medical curriculum.<br><br>More than 60% of the students rated 21 out of 38 topics as essential or important.	Strengths: High response rate; The study included a broad range of nutrition topics and compared students' perceptions with those of Faculty and GPs, providing a comprehensive view of priorities.<br><br>Limitations: Non-respondents and non-attendees could not be followed up due to ethics requirements; The timing of the survey close to final examinations may have influenced the number of topics students rated as essential or important; The study only included students who attended the Fixed Resource Session, possibly excluding those with less interest or availability.	[109] doi:10.1111/j.1445-5994.1996.tb02933.x
Longitudinal observational	2316 students from 16 U.S. medical schools / 87%, 78%, and 75% for the three questionnaire administrations.	Questionnaires	Nutrition counselling more relevant (72%) compared to students at ward orientation (61%) and senior year (46%)<br><br>Only 19% of students felt extensively trained in nutrition counseling by their senior year, and 17% reported frequently counseling patients about nutrition.<br><br>Female students, those consuming more fruits and vegetables, those who believed in primary prevention, and those with personal physicians who emphasized disease prevention were more likely to find nutrition counseling highly relevant.	Strengths: Large, diverse sample; Longitudinal data.<br><br>Limitations: The schools were not randomly selected, which might affect generalizability; Potential bias in self-reported behaviors and attitudes; Missing data for some variables, although multiple imputation was used to address this.	[110] https://doi.org/10.1093/ajcn/84.3.655

			Perceived relevance of nutrition counseling declined throughout medical school, especially among those intending to subspecialize.<br><br>Students who ate more fruits and vegetables and believed in their credibility if they ate a healthy diet were more likely to counsel patients about nutrition.		
Cross-sectional	114 internal medicine interns / 54%	Questionnaires	77% of interns agreed that nutrition assessment should be part of routine primary care, and 94% felt obligated to discuss nutrition with patients. However, 86% felt that most physicians were not trained to discuss nutritional issues.<br><br>Only 14% felt adequately trained in nutrition counseling, with notable deficiencies in areas like calculating BMI, analyzing food labels, and understanding specific nutritional needs related to diseases.<br><br>Interns answered 66% of the knowledge questions correctly on average. Knowledge was particularly lacking in areas related to nutrition assessment, obesity, endocrine nutrition, and cardiovascular nutrition.	Strengths: The study addresses an important gap in medical education regarding nutrition; It uses validated instruments for assessing attitudes and self-perceived proficiency; The findings provide actionable insights for improving nutrition education in medical training.<br><br>Limitations: The response rate was just over half, potentially introducing selection bias; The knowledge quiz was not previously validated, and the length of the survey might have affected the response quality.	[56] https://doi.org/10.1080/07315724.2008.10719702.
Cross-sectional	3267 medical students / 28.6%	23-item survey questionnaire distributed online	The mean satisfaction score was 4.7 out of 10, indicating low satisfaction.<br><br>Students felt comfortable counseling on basic nutrition concepts and prevention of disease but less comfortable with nutrition in disease treatment and identifying credible sources of nutrition information.<br><br>87.2% of respondents believed more time should be dedicated to nutrition education.	Strengths: The study provides a broad overview of the perceptions of many medical students across multiple institutions in Canada. It highlights the variability and inadequacies in nutrition education in medical schools, paving the way for future improvements.<br><br>Limitations: Only 9 out of 17 approached schools participated, which may limit the generalizability of the findings; The study relied on self-reported data, which may introduce bias; Differences in local curriculum integration of nutrition content were not	[111] doi:10.1139/H10-016

Cross-sectional	260 first- and second-year medical students / is not explicitly stated but was less than 100%	Questionnaires	There was a significant correlation between the amount of nutrition instruction and student satisfaction.<br>Most students (88%-93%) agreed that nutrition education should be an important aspect of their medical education.<br>Only a minority (22%-30%) felt that nutrition was effectively integrated into the curriculum.<br>A substantial increase in perceived effective integration was noted when nutrition was purposely added to the CMBM course for the class of 2008 (68%) compared to the class of 2007 (15%).<br>Most students indicated that nutrition should be integrated into both clinical and didactic studies rather than didactic only.<br>Students identified the need for more nutrition education integrated into existing courses and tailored to specific medical specialties.	accounted for, which could affect the interpretation of results.<br>Strengths: The study provided a clear assessment of medical students' perceptions regarding the integration of nutrition education in their curriculum. It included both quantitative and qualitative data, offering a comprehensive view of student opinions and suggestions.<br>Limitations: The study used a sample of convenience, which may not accurately represent the entire student population.	[112] doi:10.1097/TIN.0b013e318219318d.
Survey	125 invited, 65 responded (52%)	Online survey	80% perceived nutrition education as very important, but 78.5% felt instruction was inadequate.	Strengths: Identified specific areas of deficit. Limitations: Low response rate, self-reported data.	[138] Gomathi, K.G.; Shehnaz, S.I.; Khan, N. Is More Nutrition Education Needed in the Undergraduate Medical Curriculum?: Perceptions of graduates from a medical university in the United Arab Emirates. Sultan Qaboos Univ. Med. J. 2014, 14, e551.
Survey	133 invited, 121 responded (91%)	Online survey	Most U.S. medical schools (71%) fail to meet the recommended minimum of 25 hours of nutrition education. 36% provide less than half of the recommended hours.	Strengths: High response rate; Use of consistent survey questions over time allows for reliable comparisons.	[16] https://doi.org/10.1155/2015/357627

			Nutrition education is mostly confined to preclinical courses, averaging 14.3 hours, with less emphasis during clinical training, which averages only 4.7 hours.<br><br>Only 8 schools reported 40 or more hours of nutrition education, integrating courses with clinical practice.<br><br>Schools actively using the Nutrition in Medicine (NIM) online materials reported more nutrition instruction hours (22.1 vs. 17.4 hours).	Limitations: Self-reported data, variations in interpretation of 'nutrition education'.	
Cross-sectional design	351 students, 52% responsive rate.	Survey	Students believed incorporating nutrition care into practice is important.<br><br>Students were confident in skills related to nutrition in health and disease but less confident in skills related to general food knowledge.<br><br>Greater quantity and quality of nutrition education received was associated with greater self-perceived skills in providing nutrition care to patients but not with attitudes towards incorporating nutrition care into practice.<br><br>There was a limited relationship between perceptions of quantity and quality of nutrition education and students' attitudes towards nutrition care.	Strengths: Use of two previously validated tools to guide the development of the questionnaire enhances confidence in findings. Limitations: Low response rate.	[113] Crowley, J.; Ball, L.; Han, D.Y.; Arroll, B.; Leveritt, M.; Wall, C. New Zealand medical students have positive attitudes and moderate confidence in providing nutrition care: a cross-sectional survey. J Biomed Educ 2015, 2015, 259653.
Cross-sectional	122 interns / 42%	Survey	Only 29% of interns felt that their medical school education had sufficiently exposed them to clinical nutrition.<br><br>Interns who felt prepared reported an average of 4 ± 3.4 weeks of training in medical school,	Strengths: Large and diverse sample size representing multiple academic institutions; Timing of survey captured perceptions of interns midway through their first year, balancing recent medical school experiences with some clinical exposure.<br><br>Limitations: Recall and non-responder bias inherent in self-reported surveys; Potential variability in the	[134] https://doi.org/10.1177/0148607115571016

			while unprepared interns reported an average of 2 ± 2.6 weeks (P = .02).<br><br>Interns with prior graduate training in nutrition almost exclusively reported that medical school training was insufficient (94%, P = .02).<br><br>The only significant factor associated with perceived preparedness was the number of weeks of nutrition training during medical school (P = .03).<br><br>Most interns (71%) felt unprepared to handle cases requiring knowledge of clinical nutrition, especially in situations where nutrition therapy is critical.<br><br>Only 60% correctly identified the best nutritional support method for a resuscitated trauma patient; 46% recognized the role of early enteral feeding in reducing pneumonia risk.	quality and intensity of nutrition education across different medical schools; Self-reported data on weeks of training may reflect individual interest rather than actual exposure.	
Cross-sectional study	272 first to fourth-year students / 46% response rate for first and second-year students and 24% response rate for third and fourth-year students	For first and second-year students, the survey was conducted in a lecture theatre using TurningPoint 5 Interactiv	Most preclinical students (79%-99%) indicated that understanding nutritional issues related to specific diseases (e.g., cardiovascular disease, type 2 diabetes, coeliac disease, and renal impairment) is important.<br><br>Confidence in demonstrating nutrition knowledge was low among preclinical students (26%-41%), but it increased for clinical students in years 3 and 4 (26%-81%). However, confidence in knowledge related to medicolegal issues, respiratory disease, nutritional guidelines, and nutrition assessment remained low (<40%) even in clinical years.	Strengths: The study included medical students across all four years of the postgraduate course, providing a comprehensive overview of perceptions across different stages of medical education; By evaluating both the perceived importance and confidence in nutritional knowledge, the study highlights specific areas for curriculum improvement.<br><br>Limitations: The response rates were less than 50%; The study assessed self-perceived confidence and importance rather than actual knowledge and competence, which may not accurately reflect students' true abilities; The study focused on a selected number of medical conditions, potentially	[114]<br>doi:10.1016/j.puhe.2016.08.019

		e PowerPoint® software.<br>For third and fourth-year students, the survey was distributed via email with a link to an online anonymous survey (Qualtrics)	Students reported higher confidence in the dietary management of type 2 diabetes, cardiovascular disease, and coeliac disease, but lower confidence in conditions where diet is secondary to other treatments (e.g., respiratory disease)	overlooking other important areas of nutritional management.	
Cross-sectional survey	134 to 147 first-year medical students, 32% to 61% responsive rate.	Online survey	While 59%-93% of students knew the recommended daily servings for fruit, and 61%-84% knew the recommendations for vegetables, only 40%-46% met the fruit intake guidelines and 12%-19% met the vegetable intake guidelines.<br>Few students knew the guidelines for daily salt intake (8%-26%), but a majority avoided adding salt at the table (73%-85%).<br>he study highlighted a significant gap between nutrition knowledge and dietary practices among medical students, suggesting the need	Strengths: Longitudinal data collection over four years provided a comprehensive view of trends in knowledge and practices; The study used a standardized survey method, allowing for consistent data collection across different cohorts.<br>Limitations: Self-reported data on dietary practices may be subject to bias; Changes in survey question wording over the years may have affected comparability of responses across cohorts.	[115] Perlstein, R.; McCoombe, S.; Macfarlane, S.; Bell, A.C.; Nowson, C. Nutrition Practice and Knowledge of First-Year Medical Students. J Biomed Educ 2017, 2017, 5013670.

			for more effective educational strategies to bridge this gap.		
Qualitative study	48 medical students, 14 residents / 51%	Focus groups and one-on-one interviews	Nutrition education was poorly integrated into the medical school curriculum.<br>Both medical students and physicians felt the nutrition education they received was inadequate.<br>Students observed very little nutrition counselling during their shadowing experiences, and the information was often outdated or incorrect.<br>Residents felt ill-prepared to offer nutrition counselling and desired further education in this area.	Strengths: Provided insights directly from medical students, residents, and physicians regarding the state of nutrition education; Highlighted the perceived inadequacies and the need for improved training in nutrition counselling.<br>Limitations: Participation rate was 51%, and higher participation might have yielded different results.	[128] Danek, R.L.; Berlin, K.L.; Waite, G.N.; Geib, R.W. Perceptions of nutrition education in the current medical school curriculum. Fam. Med. 2017, 49, 803–806.
Qualitative study	17 Medical students	Web-based survey	Most students were familiar with obesity medicine through various courses, primarily Biochemistry or Nutrition (94%), Endocrinology (82%), and Wellness (70%).<br>There was significant variability in student knowledge about basic skills such as measuring waist circumference.<br>About half of the students did not feel knowledgeable about recommending weight loss treatments.<br>Most students did not feel prepared to provide interventions for patients with different stages of obesity, psychosocial issues, or obesity-related comorbidities.	Strengths: The study identified specific gaps in obesity medicine education, which can inform curriculum improvements; Student perspectives were considered, providing a comprehensive view of the educational needs.<br>Limitations: Small sample size; The use of convenience sampling and reliance on publicly available contact information could introduce bias; The findings are based on self-reported data, which may be subject to response biases.	[116] doi:10.2196/mededu.7361.
Cross-sectional study	1507 medical students enrolled	Paper-based	87% of respondents believed that high-risk patients should be routinely counselled on	Strengths: The study highlights the recognition among future physicians of the importance of nutrition in patient care, indicating a positive	[117] doi:10.1080/07315724.2017.1333928

	across all 4 years / 61.6%	questionnaire	nutrition, and 70% felt that nutrition counselling should be routine practice.<br><br>86% agreed that performing some level of nutritional assessment with every patient is important, but only 38% felt that asking for a food diary or other measure of dietary intake was important.<br><br>Few students felt confident in their knowledge of nutrition-based treatments, with only 3% stating they know enough to treat a patient using nutrition-based treatments.	attitude towards integrating nutrition into standard care practices.<br><br>Limitations: There was a lower response rate from third- and fourth-year students due to their geographical dispersion during clinical rotations; The survey did not allow for qualitative data collection, which could have provided deeper insights into the students' responses.	
Cross-sectional study	215 clinical level medical students, 96% responsive rate	Paper-based, self-administered questionnaire.	92% of students considered nutrition education relevant to their future practice.<br><br>The majority were dissatisfied with the amount of time dedicated to nutrition education (70%), integration of nutrition into organ-system based modules (62%), inclusion of nutrition materials to promote independent study (62.8%), and the nutrition course content (59%).<br><br>Only 22.2% felt adequately prepared by their current nutrition education to provide nutrition care in general practice.<br><br>Satisfaction with current education in nutrition was positively related to students' preparedness to provide nutrition care.	Strengths: The study used a previously validated survey instrument; It covered a significant percentage of the target population (96% response rate); The content validity was reviewed by experts in nutrition and health professions education.<br><br>Limitations: The cross-sectional nature of the study limits the ability to establish causality; Findings may be subject to social desirability bias, although self-critical responses from students suggest this was minimized.	[135] https://doi.org/10.1016/j.hpe.2017.02.003
Cross-sectional study	342 medical students (second, third, and fourth years), 26% responsive rate	Online survey using Opinio™ software	Mean satisfaction with the nutrition curriculum was 2.9 ± 0.81 on a five-point Likert scale.<br><br>Highest satisfaction in nutrition assessment (mean rating of 3.98 ± 0.89), lowest in strategies to address food security (mean rating of 2.4 ± 0.95).	Strengths: Provides actionable recommendations based on student feedback; Uses a comprehensive survey instrument adapted from a previous validated study.<br><br>Limitations: Low response rate (26%) which may limit the generalizability of the findings; Higher proportion of second-year students responded compared to other years, potentially skewing	[118] doi:10.1093/jcag/gwy043

			Significant difference found in understanding the role of registered dietitians, with Med-4 students more confident than Med-2 students.<br>High agreement on the importance of nutrition in disease prevention and management (all students agreed or strongly agreed).<br>Students recommended a longitudinal nutrition program, inclusion of dietitians as educators, and provision of evidence-based resources. 79% agreed more nutrition instruction is needed.	results; The study relied on self-reported data, which may introduce response bias.	
Qualitative study	23 students from the 5th to final year. Response rate is not explicitly mentioned, but all 23 students contacted participated.	Semi-structured individual interviews.	Doctors should provide basic nutrition care, collaborate with nutritionists/dieticians, monitor nutrition care progress, and advocate for nutrition care.<br>Students felt unconfident in their ability to provide nutrition care.<br>Perceived their nutrition education as inadequate due to limited training.<br>Lack of priority for nutrition education in the curriculum.<br>Insufficient faculty trained in nutrition.<br>Poor application of nutrition science to clinical practice.<br>Inadequate collaboration with nutrition professionals.<br>Increase lectures, tutorials, and self-study time for nutrition.<br>Integrate nutrition as a theme throughout the entire curriculum.	Strengths: Provided rich, detailed insights from the students' perspectives; Offers actionable strategies to improve nutrition education.<br>Limitations: Non-probability and convenience sampling may have led to the inclusion of students with a particular interest in nutrition; Focused on students' perceptions without evaluating the impact of improved nutrition education on clinical outcomes.	[119] doi:10.1186/s12909-018-1130-5

			Raise awareness about the importance of nutrition education.<br><br>Review and revise the curriculum to incorporate nutrition.<br><br>Involve nutrition/dietician specialists in medical education.		
Qualitative study	13 second-year students and 35 third-year students / 14% and 12.3%, respectively	Semi-structured focus groups and interviews	Students acknowledged the role of doctors in providing nutrition care, especially for lifestyle-related diseases.<br><br>Identified barriers included lack of time, insufficient resources, inadequate nutrition education, and limited confidence.<br><br>Students had variable nutrition knowledge and found difficulty translating theoretical knowledge into clinical practice.<br><br>Confidence in applying nutrition-related skills was generally low. Students suggested more practical engagement with dietitians.	Strengths: Provides in-depth understanding of medical students' perspectives on nutrition education; Highlights gaps in current medical curricula regarding nutrition care.<br><br>Limitations: Low response rates and potential sampling bias due to the voluntary nature of participation. Qualitative methods do not provide objective measures of nutrition knowledge but offer insights that quantitative methods might not.	[129] https://doi.org/10.3390/nu12030598
Qualitative research	14 postgraduate medical students	Individual semi-structured qualitative interviews	Medical students perceived nutrition to be a foundational and central component of ideal medical management, especially for chronic diseases.<br><br>Students experienced variability in the importance placed on nutrition by different educators and healthcare professionals.<br><br>Students acknowledged the role of various healthcare professionals, such as dietitians, in providing nutrition care and recognized the collaborative nature of nutrition management.	Strengths: The study used a theory-informed approach to data collection and analysis; It included a diverse sample of students across different stages of their medical education; The iterative and team-based coding approach ensured thorough data analysis.<br><br>Limitations: The study might have been influenced by the societal attitudes towards nutrition, which could vary across different regions and times.	[136] Martin, S.; Sturgiss, E.; Douglas, K.; Ball, L. Hidden curriculum within nutrition education in medical schools. BMJ Nutr. Prev. Health 2020, 3, 18.
Narrative synthesis	853 participants, which included	Surveys distribute	Over 90% of respondents agreed on the importance of nutrition in health and a doctor's	Strengths: The study combined data from multiple sources, providing a broad view of the current state	[120] Macaninch, E.; Buckner, L.; Amin, P.; Broadley, I.; Crocombe,

	both medical students and doctors	d via email and social media.<br><br>Module evaluations at a single UK medical school.<br><br>One-on-one interviews and Visual Analogue Scale (VAS) questionnaires.	role in nutritional care. However, there was less desire for additional nutrition education among doctors (85%) and medical students (68%).<br><br>Most respondents felt their nutrition training was inadequate, with over 70% reporting less than 2 hours of nutrition education. Face-to-face training was preferred over online methods.<br><br>Confidence in nutrition knowledge and skills was low among both medical students and doctors. Only 26% of doctors felt confident in their nutrition knowledge, and 74% gave nutritional advice less than once a month due to lack of knowledge, time, and confidence.	of nutrition education in medical training across the UK; t utilized both qualitative and quantitative methods, offering a rich analysis of the perspectives of medical students and doctors at various stages of their education and careers.<br><br>Limitations: Respondents were likely those with a pre-existing interest in nutrition, potentially skewing the results. The use of different survey designs and evaluation techniques reduced comparability between studies. The small and self-selected sample size limits the generalizability of the findings.	D.; Herath, D.; Jaffee, A.; Carter, H.; Golubic, R.; Rajput-Ray, M. Time for nutrition in medical education. BMJ Nutr Prev Health 2020, 3, 40.
Cross-sectional questionnaire study	1669 undergraduate medical students / 11%	Online questionnaire	Students' perceived relevance of Culinary Medicine knowledge and its application to future clinical practice.<br><br>83% of students felt learning Culinary Medicine is important for their future clinical practice.<br><br>56% felt unable to take a dietary history.<br><br>73% were dissatisfied with the quality, and 78% were dissatisfied with the quantity, of existing medical school teaching relevant to Culinary Medicine.	Strengths: The questionnaire was piloted to confirm readability and ensure questions addressed the study aim; Independent coding during qualitative thematic analysis improved the breadth and quality of interpretations.<br><br>Limitations: Low response rate (11%) limits the generalisability of results.<br><br>Students with prior understanding and/or interest in Culinary Medicine were more likely to complete the questionnaire, potentially biasing the results.	[121] doi:10.1136/bmjopen-2019-036410.

Cross-sectional	100 responses analysed (72% resident surgeons, 28% faculty surgeons)	Online survey	90.2% of residents and 85.7% of faculty surgeons reported having received nutritional education.<br><br>78% of respondents utilized patient nutrition regularly, with 54% managing nutritional care weekly or daily.<br><br>65% of respondents reported challenges in managing patient nutritional needs, particularly in determining specific nutritional formulas and managing various nutritional delivery routes (oral, enteral, and parenteral).<br><br>A significant number of residents received formal nutritional education, whereas faculty surgeons relied more on informal modalities such as clinical experience and self-education.<br><br>86% agreed that additional nutritional education during training would assist with patient care.	Strengths: The study highlights a critical gap in the nutritional education of surgeons, with broad implications for patient care and outcomes; Uses a comprehensive survey to gather detailed insights from a relevant sample (surgery residents and faculty surgeons).<br><br>Limitations: Responses may be affected by participants' memory of their education; Limited number of responses may affect the generalizability of the findings; The pandemic may have influenced response rates and the generalizability of the results due to changes in hospital operations.	[122] doi:10.1177/0003134820971621
Survey-based research	200 medical students and 175 residents	Online Survey	A significant number of trainees perceived inadequate nutrition training during medical school.<br><br>Positive interest in including whole-food, plant-based nutrition-focused curriculum in medical training was reported.<br><br>Respondents were more likely to recommend a whole-food, plant-based diet if they were familiar with its health benefits.<br><br>Barriers such as feasibility and personal dietary preferences were highlighted as challenges to recommending a whole-food, plant-based diet.	Strengths: The study fills a gap in the literature regarding the acceptability of WFPB diet-focused curriculum in medical education; Use of both quantitative and qualitative data provides a comprehensive understanding of perceptions and attitudes.<br><br>Limitations: Low overall response rate, which may affect the generalizability of the findings; The study focused specifically on a whole-food, plant-based diet, excluding other dietary patterns, which may have biased the results towards perceiving whole-food, plant-based as superior without comparative analysis.	[123] doi:10.1177/1559827620988677

Cross-sectional	30 medical students from various grades and 6 faculty members	Individual, semi-structured interviews	Participants emphasized the significant role of nutrition in medical care and health, viewing it as essential for prevention and treatment of various medical conditions.<br><br>Both students and faculty believed that doctors should promote good nutrition and require adequate education to provide general nutrition information to patients. Barriers such as limited time, inadequate nutrition knowledge, and inconsistent emphasis on nutrition topics were identified.<br><br>While the theoretical education on nutrition was considered good, the practical application and emphasis on nutrition in clinical practice were seen as inadequate. Nutrition topics were not consistently emphasized across all subjects.<br><br>There was a strong interest among students for more practical nutrition education, including self-care and interactive learning methods such as case studies and role plays.	Strengths: Inclusion of diverse perspectives from both Czech and English program students and faculty members with different expertise and clinical experience; Standardized interview guides and trained facilitators ensured consistent data collection.<br><br>Limitations: Convenience sampling may not have captured a representative sample of the student and faculty population, potentially biasing the findings; Translation of materials and transcripts might have led to the loss of subtle information.	[124] doi:https://doi.org/10.1016/j.clnesp.2021.11.011
Cross-sectional	2252 students	Anonymous and non-validated questionnaire	Overall, 9.9% of respondents indicated a high likelihood (score ≥8 out of 10) of choosing Endocrinology and Nutrition (E&N).<br><br>Only 2.6% considered E&N as their first choice (score 10 out of 10), with a higher proportion among non-Spanish university candidates (5.5%) compared to Spanish university candidates (1.4%).<br><br>Logical pathophysiological basis (54%), dynamic and varied work (27%).<br><br>Pre-graduate teaching had a very positive influence on 37.9% of those who considered E&N as their first choice.	Strengths: Large sample size; Wide representation<br><br>Limitations: Non-validated questionnaire; The data is based on self-reports, which could be influenced by personal biases or inaccurate self-assessment.	[125] https://doi.org/10.1016/j.endien.2023.04.004

Qualitative research study	30 medical students and residents.	Semi-structured interviews	Hypothalamic-pituitary disorders and diabetes mellitus were the most attractive areas, with clinical nutrition added for those considering E&N as their first choice.<br><br>Medical students and residents expressed mixed feelings about their nutrition education, highlighting gaps between their personal beliefs about nutrition and the formal education they received.<br><br>Hands-on, system-based, epistemological, and multidisciplinary approaches were suggested to improve nutrition education.<br><br>Identified barriers included a lack of practical training, insufficient integration of nutrition into the overall curriculum, and perceived undervaluation of nutrition by medical schools	Strengths: The use of qualitative interviews provided in-depth insights into the personal experiences and expectations of medical students and residents.<br><br>Limitations: The sample size was relatively small; The study relied on self-reported data.	[126] Thircuir, S.; Chen, N.N.; Madsen, K.A. Addressing the Gap of Nutrition in Medical Education: Experiences and Expectations of Medical Students and Residents in France and the United States. *Nutrients* **2023**, *15*, 5054.
