# Supplementary material for: Nutrition and Physical Activity Education in Medical School: A Narrative Review
Source: Nutrients. 2024 Aug 22;16(16):2809. doi: 10.3390/nu16162809 (PMC11357297; doi:10.3390/nu16162809)
Supplement: Supplementary file 1 [file nutrients-16-02809-s001.zip › Table S4.pdf]

Table S4. Detailed analysis of medical students' perceptions and knowledge regarding physical activity (PA) education across multiple institutions, highlighting participation rates, assessment methods, key findings, and identified strengths and limitations of the studies reviewed

Study type	Target size/Responses rate	Collection Method	Key Findings	Strengths/Limitations	Reference
Cross-sectional	Total of 6797 students participated<br>1954 males<br>4833 females	Data from school database for participant characteristics<br>Physical fitness tests conducted in a gymnasium:<br>BMI measurement<br>Vital capacity index<br>Sidestep test<br>Standing long jump	Female students had a higher rate of normal weight and performed better in most physical fitness tests compared to male students.<br>Male obesity rate was five times higher than female obesity rate.<br>In the malnutrition and normal weight groups, both male and female students had higher pass rates in the three physical fitness tests compared to the obese group.<br>The study found that male students might be spending more time using computers, reducing their physical activity time.	Strengths: Large sample size providing significant data on gender differences in physical fitness; Use of standardized physical fitness tests ensuring consistent data collection.<br>Limitations: Cross-sectional nature of the study limits causality conclusions; Self-reported data on physical activity may introduce bias.	[141] doi:10.5539/gjhs.v7n1p220
Cross-sectional	87 medical students (29 males and 58 females) aged between 18-25 years / Not explicitly mentioned, but all 87 students participated.	The study utilized the International Physical Activity Questionnaire (IPAQ) to assess physical activity (PA) levels and Metabolic Equivalent Task (MET) scores. Participants provided detailed history of their PA, including walking, moderate-intensity activities, and vigorous-intensity activities.	MET Score Distribution: 40% of students fell into the low PA group, 47% into the moderate PA group, and 13% into the high PA group.<br>Conclusion: Physical activity was found to be very low among medical students based on the MET scores calculated using the IPAQ.	Strengths: Used a standardized and internationally recognized tool (IPAQ) for assessing PA; Provided specific MET calculations for different types of physical activities, which allows for a detailed analysis of PA levels.<br>Limitations: The study was conducted on a small sample size limited to a single institution, which may not be representative of the broader population of medical students; Self-reported data via questionnaires may be subject to recall bias and inaccuracies.	[142] Ashok, P.; Kharche, J.S.; Raju, R.; Godbole, G. Metabolic equivalent task assessment for physical activity in medical students. Natl J Physiol Pharm Pharmacol 2017, 7, 236.

				Did not account for potential environmental factors or individual skill levels that could affect MET calculations.	
Cross-sectional	All third-year medical students at the Dunedin School of Medicine, University of Otago (n=237) / 99%	Students completed a 15- to 20-minute paper-based questionnaire during tutorial classroom time. The questionnaire assessed knowledge, skills, and attitudes toward exercise advising, as well as personal PA habits	Most students were aware of the benefits of PA and current New Zealand PA guidelines, but only 19% knew the recommended combination of duration, intensity, and frequency of activity for healthy adults. Awareness of American College of Sports Medicine guidelines for chronic conditions was low (2%).<br><br>PA advising was perceived as a high priority (79%), but only 37% felt confident in providing PA advice. Students rated the importance of PA advising high (4.7/6) but felt only moderately competent (3.1/6).<br><br>Most students (79%) were physically active, meeting minimal PA guidelines. Regularly active students felt more confident in providing PA advice.	Strengths: High response rate (99%) enhances the reliability of the findings; Comprehensive assessment of students' knowledge, attitudes, and personal habits regarding PA.<br><br>Limitations: Cross-sectional design: Limits causal inferences; Self-reported data: Potential for bias and inaccuracies; Timing of assessment: Third-year students were surveyed before exposure to specific PA advising training.	[146] Mandic, S.; Wilson, H.; Clark-Grill, M.; O'Neill, D. Medical Students' Awareness of the Links between Physical Activity and Health. Montenegrin J. Sports Sci. Med. 2017, 6, 5–12.
Cross-sectional	1356 final year medical students from seven UK medical schools / 11.6%	An online Google survey consisting of nine key questions was sent via email and posted on social media groups for final year medical students. The survey was live for ten days between 9 December 2018 and 19 December 2018	52% of the participants were unaware of the current exercise guidelines in the UK.<br><br>80% of the participants stated they had not received training in lifestyle medicine over the last two years.	Strengths: The study highlights a significant gap in knowledge among final year medical students regarding physical activity guidelines and the lack of training in lifestyle medicine, which is crucial for addressing chronic health conditions.<br><br>Limitations: The response rate was relatively low (11.6%), which might limit the generalizability of the findings; The survey relied on self-reported data, which could be subject to response bias.	[147] https://doi.org/10.1136/bmjsem-2019-000518

			48.1% were unacquainted with motivational interviewing.<br><br>76% expressed a desire for more lifestyle medicine teaching to be incorporated into the medical school curriculum.		
Cross-sectional	124 medical interns / 83.1%	Data was collected using a pre-tested, semi-structured questionnaire administered to the interns who were posted in the Department of Community Medicine. The questionnaire included demographic details, details about physical activity, height, and weight. The data was analysed using Microsoft Excel 2010 and SPSS version 21.	Majority of the interns (64.1%) had a normal body mass index (BMI).<br><br>Non-exercise physical activity was more common in female interns, while exercise physical activity was more prevalent among male interns.<br><br>81 (78.6%) of the interns were physically active.<br><br>Statistically significant differences were observed in non-exercise physical activities like preparing food, washing, ironing (more in females), and exercise physical activities like playing cricket and football (more in males).	Strengths: The study highlights the importance of physical activity among future doctors and provides insight into gender differences in physical activity patterns among medical interns.<br><br>Limitations: Small sample size, reliance on self-reported data which may be subject to recall bias, and the study being conducted in a single medical college, limiting the generalizability of the findings.	[143] Ramkumar, B.; Aswathy, R.; Pavithra, A. Study on practice of physical activity among medical interns in a private medical college hospital in Chennai. 2019.
Cross-sectional	480 medical students	The data was collected using a 19-question electronic survey distributed via Research Electronic Data Capture (REDCap, Vanderbilt University, Nashville, TN) from August 2019 to May 2020. The survey was distributed via email to medical student members of the American Medical Society of Sports Medicine (AMSSM), primary contacts at AMSSM Charter Medical Schools, and	Students are more comfortable discussing overall exercise benefits than exercise testing, exercise prescription, and exercise physiology.<br><br>There is more exposure to general PA guidelines related to overall PA duration than strength training.<br><br>No significant difference between allopathic and osteopathic schools	Strengths: Broad representation of medical schools; Detailed breakdown of comfort levels in various PA-related topics.<br><br>Limitations: Potential response bias due to lack of incentivization, which may have led to overrepresentation of students interested in PA; Non-representative sample due to higher responses from certain medical schools; Recall bias as respondents were asked to report on past education; Survey timing might have led to underestimation of current year PA education	[145] https://doi.org/10.22454/primer.2021.249084

		through social media channels such as Facebook and GroupMe.	regarding familiarity with PA guidelines.<br><br>Students from smaller institutions (class sizes under 200) reported increased familiarity with National Physical Activity Guidelines.<br><br>Despite PA education improvements, students feel unprepared to apply their knowledge in clinical settings and remain unaware of national PA guidelines.	hours; Lack of weighted responses due to a small number of respondents from many schools; Use of binary assessment for comfort levels instead of a Likert scale.	
Cross-sectional	107 pre-clinical medical students / 37.4%	An online survey was administered using Qualtrics survey software. Participants were invited through lectures, flyers, and social media, and they completed the survey in September 2017. The survey included questions about demographics, past-week physical activity behaviour, understanding of physical activity messages, and perceptions of the role of GPs in promoting physical activity	92% of students were moderately or highly active in the past week.<br><br>Knowledge of key physical activity messages was moderate (mean score of 3.6 out of 5), with understanding varying significantly across different messages.<br><br>High agreement among students that discussing the benefits of physical activity is part of the GP's role (mean score of 4.7 out of 5).<br><br>Moderate agreement on having received training in the health benefits of physical activity (mean score of 3.1) and physical activity counseling (mean score of 3.2).<br><br>Low satisfaction with the training received (mean score of 2.5)	Strengths: Addresses a gap in understanding current medical students' knowledge and attitudes toward physical activity promotion; Provides insight into future medical practitioners' preparedness to promote physical activity.<br><br>Limitations: Small sample size from a single university, limiting generalizability; Potential bias due to self-selection of physically active students; Use of self-reported data, which may be subject to recall and social desirability biases	[144] https://doi.org/10.1186/s12909-022-03695-y

Cross-sectional	793 medical students / 54.5%	A link to the online survey was sent to all medical students in their first through fourth years of study at the University of Miami Leonard M. Miller School of Medicine via various student email listservs. Students received additional reminders one and two weeks after the initial email asking them to complete the survey. Prior to starting the survey, medical students were asked to read a brief introduction on the study outlining their risks and benefits to participating and provide their informed consent.	Importance of Training: Nearly all medical students (92.4%) considered training in chronic disease prevention important. Specific topics rated as "very important" or "important" included nutrition (90%), obesity (90%), tobacco/smoking cessation (90.1%), and physical activity (79%).<br><br>Lack of Awareness: Only a quarter of students were "very aware" of public health prevention programs, and just 5% could correctly list an evidence-based chronic disease program.<br><br>Desire for Applied Experiences: Medical students desired greater exposure to primary prevention programs as part of their training, with a significant portion expressing interest in applied experiences outside of the clinical setting.<br><br>Differences by Demographics: Female students and those in the MD/MPH program rated the importance of training higher compared to their counterparts. Students earlier in their medical education valued training in chronic disease prevention more than those further along in their training.	Strengths: The survey was easily administered and achieved a high response rate, which is representative of the medical school student population in terms of year in school, sex, and age, Provides a framework for similar studies to be conducted at other institutions.<br><br>Limitations: Lack of external generalizability as the study was conducted at a single institution; Participation was voluntary and anonymous, introducing potential selection bias; More students with positive opinions towards chronic disease prevention training may have responded; Overrepresentation of MD/MPH students compared to MD students; Some key terms were not explicitly defined, which may have affected students' responses.	[148] https://doi.org/10.1186/s12909-023-04044-3
